# Direct Flavonoid-Focused Chemical Comparison among Three *Epimedium* Plants by Online Liquid Extraction–High Performance Liquid Chromatography–Tandem Mass Spectrometry

**DOI:** 10.3390/molecules26061520

**Published:** 2021-03-10

**Authors:** Xia Xu, Ting Li, Ke Zhang, Yan Cao, Li Liu, Shilin Zhang, Pengfei Tu, Yuelin Song, Yunfang Zhao, Jun Li

**Affiliations:** 1 Modern Research Center for Traditional Chinese Medicine, School of Chinese Materia Medica, Beijing University of Chinese Medicine, Beijing 100029, China; Xkilly1119@163.com (X.X.); 18201353145@163.com (T.L.); zk_5353@163.com (K.Z.); cyan199409@163.com (Y.C.); pengfeitu@163.com (P.T.); syltwc2005@163.com (Y.S.); 2 Guizhou Hanfang Pharmaceutical Co., Ltd., Guiyang 550014, China; dcxliuli@163.com (L.L.); zhang_shi_lin2021@163.com (S.Z.)

**Keywords:** Epimedii Herba, online liquid extraction, flavonoids, neutral loss of rhamnosyl residue, mass defect filtering

## Abstract

It is usually a tedious task to profile the chemical composition of a given herbal medicine (HM) using high performance liquid chromatography–tandem mass spectrometry (LC–MS/MS) due to the time-consuming sample preparation and laborious post-acquisition data processing procedures. Even worse, some labile compounds may face degradation risks when exposed to organic solvents for a relatively long period. As one of the most popular HMs, the promising therapeutic benefits of Epimedii Herba (Chinese name: *Yinyanghuo*) are well defined; however, the chemical profile, and in particular those flavonoids that have been claimed to be responsible for the efficacy, remains largely unknown. Attempts are devoted here to achieve direct LC–MS measurement and efficient post-acquisition data processing, and chemome comparison among three original sources of Epimedii Herba, such as *Epimedium sagittatum* (Esa), *E. pubescens* (Epu), and *E. koreanum* (Eko) was employed to illustrate the strategy utility. A home-made online liquid extraction (OLE) module was introduced at the front of the analytical column to comprehensively transfer the compounds from raw materials onto the LC–MS instrument. A mass defect filtering approach was programmed to efficiently mine the massive LC–MS dataset after which a miniature database was built involving all chemical information of flavonoids from the genus *Epimedium* to draw a pentagonal frame to rapidly capture potential quasi-molecular ions (mainly [M–H]^−^). A total of 99 flavonoids (66 in Esa, 84 in Eko, and 66 in Epu) were captured, and structurally annotated by summarizing the mass fragmentation pathways from the mass spectrometric data of authentic compounds and an in-house data library as well. Noteworthily, neutral loss of 144 Da was firstly assigned to the neutral cleavage of rhamnosyl residues. Significant species-differences didn’t occur among their chemical patterns. The current study proposed a robust strategy enabling rapid chemical profiling of, but not limited to, HMs.

## 1. Introduction

A given herbal medicine (HM) is usually recognized as a complicated compound pool, resulting in a dramatic technical challenge for in-depth chemical profiling, even for a single chemical plant family-focused characterization [1,2,3,4]. LC–high resolution MS has been widely favored as a fit-for-purpose analytical tool [5] for the chemical characterization of HMs owing to the combination of the separation potential of LC and the structural annotation ability of MS. Although both LC or MS instrumentation have witnessed rapid progress in recent years, there are still two technical obstacles, namely direct analysis and efficient post-acquisition data mining, to rapidly obtain reliable snapshots of chemical profiles.

Labile compounds widely exist in HMs and these compounds may face degradation risks during time-consuming sample preparation procedures because of their exposure to organic solvents, light, and high temperatures for a relatively long period. Consequently, direct analysis is of great extreme importance to draw the real picture of chemical patterns. Most HMs are solid matrices and the chemical compounds are usually distributed within the plant cells. Generally, liquid extraction is the best course to transfer compounds-of-interest from inside HM matrices to the solvent, and this procedure can be affected by several factors, such as the nature of the solvent, temperature, time duration, pressure, and so on [6,7]. It is still challenging to achieve efficient extraction due to the prerequisite of compatibility between the extraction solvent and the LC mobile phase. Fortunately, a smart online liquid extraction (OLE) module has been configured allowing direct analysis of solid matrices, where aqueous acetonitrile and even water, are usually employed as the extraction solvents because the pressurized warm solvent exhibits lower viscosity and polarity and is able to efficiently extract less polar compounds [8,9,10,11,12,13,14]. The hyphenation of this OLE module with conventional LC–MS might offer the desired opportunity for direct analysis of HMs.

Accompanying the quick development of MS equipment, the resulting datasets are becoming more and more complicated, resulting in labor- and time-intensive data processing tasks. Mass defect is defined as the difference between the exact mass and the nominal mass of a given compound. Regarding most natural products, oxygen and hydrogen atoms primarily account for the mass defect, whereas carbon atom cannot provide any contribution. Compounds belonging to an identical chemical family usually share the same scaffold, resulting in similar mass defect patterns, despite quite different molecular weights among homologues. A robust strategy, namely mass defect filtering (MDF), has therefore been proposed to accelerate information-of-interest mining from the massive datasets via capturing the ions, usually quasi-molecular ions ([M–H]^−^ or [M+H]^+^), with a rectangle, prismatic, or pentagonal frame [15]. To achieve reliable information filtering, prior knowledge such as phytochemical studies and the mass defect patterns of the concerned chemical cluster, are highly desirable. In the current study, an in-depth summary of the chemical components from the entire genus, not only the targeted species, is conducted to aid the MDF procedure.

Epimedii Herba (Chinese name: *Yinyanghuo*), consisting of the dried leaves of several *Epimedium* plants [16], has been widely utilized in the clinic for the treatment of a variety of disorders, such as erectile dysfunction, seminal emission, chronic musculoskeletal pain, infertility, urinary frequency, urinary incontinence, chronic impediment diseases, hypertension, hemiplegia following a stroke, polio, coronary sclerosis, angina pectoris, palpitation, chronic bronchitis, and leukopenia [17]. This precious HM is also able to improve the life-quality of patients undergoing maintenance hemodialysis [18]. Flavonoids, notably those bearing isopentenyl substituents, have been identified as playing primary roles in regards to the pharmacological activity spectrum [19,20,21,22]. However, direct, rapid, and in-depth chemome comparison hasn’t been conducted for the different original sources of this well-known HM, resulting in a significant obstacle for further exploitation. Therefore, Epimedii Herba was employed as a proof-of-concept example to illustrate the utility of OLE-LC–MS complemented by the MDF approach.

## 2. Results and Discussion

### 2.1. Extraction and Elution Program Optimization

The extraction efficacy is jointly governed by the solvent, temperature and extraction duration, along with the pressure that is primarily determined by the flow rate. On the basis of some preliminary assays, the combination of water and acetonitrile (ACN) was found to be the most suitable solvent choice in comparison of aqueous methanol. After careful evaluation of different mixtures ranging from 0–20% aqueous ACN (step size as 5%), 10% aqueous ACN was ultimately employed. When formic acid (0.1%, *v*/*v*) was deployed as an additive, the overall extraction yield was significantly improved. Extraction temperature (actually the column oven temperature) values of 50, 55, 60, 65, 70, 75, and 80 °C were compared and extraction durations from 3–7 min (step-size of 1 min) were assessed. As a result, the best choices were 75 °C and 5 min. Moreover, attention was paid to the flow rate optimization. A flow rate of 0.3 mL/min was allowed by the upper pressure limitations of the LC system and enabled efficient extraction. Ultimately the extraction program described in Section 3.3 was applied for the extraction phase.

**Figure 1:**
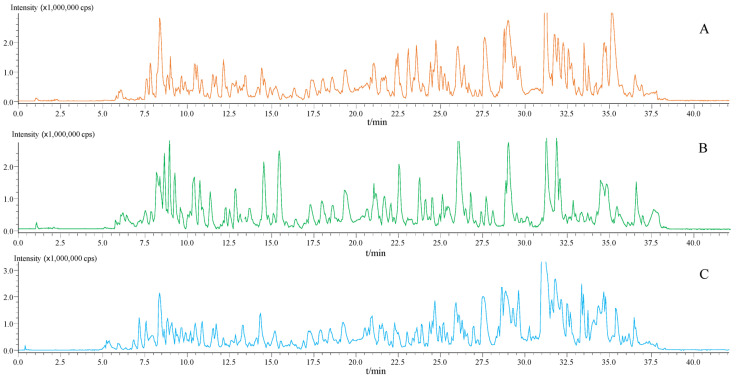
Base peak chromatograms of *E. sagittatum* ESa, (**A**), *E. koreanum* EKo, (**B**) and *E. pubescens* EPu, (**C**), in negative ion mode.

Extensive efforts were also paid onto identifying a fit-for-purpose column as well as an appropriate gradient elution program to produce a satisfactory chromatographic pattern for the *Epimedium* plants, and *Epimedium sagittatum* (Esa) powders were employed as a representative sample. A robust HSS T3 column (2.1 mm × 100 mm, 1.8 μm, Waters, Milford, MA, USA) was found to be the best choice to retain and separate most signals and also to generate better peak patterns compared to some other accessible column choices, such as the Ascentis^®^ Express F5 (2.1 mm × 150 mm, 2.7 μm, Supelco, Bellefonte, PA, USA), Capcell core ADME (2.1 mm × 150 mm, 2.7 μm, Shiseido, Tokyo, Japan), and Capcell core PFP (2.1 mm × 100 mm, 2.7 μm, Shiseido). Afterwards, the mobile phase, elution program as well as the additive were carefully assayed to advance the overall chromatographic pattern. Fortunately, the solvents (0.1% aqueous formic acid and ACN) utilized for online liquid extraction could meet the chromatographic separations requirements of most signals, and moreover, formic acid, as the additive, could improve the overall chromatographic profile. After careful optimization, a gradient elution program was defined (see Section 3.3) and the initial solvent was also the one implemented for OLE. A representative base peak chromatogram is illustrated as Figure 1A, where obviously most peaks display acceptable chromatographic behaviors.

### 2.2. Mass Defect Properties of Flavonoids

**Figure 2:**
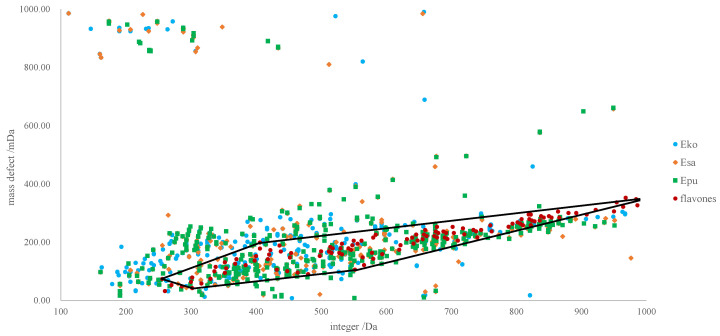
Integer parts against decimal parts of the compounds in the in-house chemical library (red solid dots) and the detected MS^1^ signals (orange diamonds for Esa, blue solid dots triangles for Eko, and green square for Epu). A five-point frame was drawn to involve all red solid dots, and only those dots in the frame were the potential flavonoids in *Epimedium* plants.

A pentagonal frame was employed for mass defect filtering (MDF), and the frame was constructed by tightly following the descriptions in the literature [15]. In brief, five compounds (a, b, c, d, and e) were applied as the five vertexes to determine the filtering scale. In theory, the values for the quasi-molecular ions (e.g., [M–H]^−^) of all organic compounds should contain integer and decimal parts, and the compounds sharing similar skeletons should have comparable mass defect patterns. Because we primarily focused on the flavonoids in *Epimedium*, only the ones belonging to this chemical family were considered for summarizing the mass defect rules. Firstly, an in-house data library that contained all the flavonoids of *Epimedium* was built. Five-point frame was subsequently constructed as the MDF scale of flavonoids for rapid screening of the MS^1^ information of Esa, *E. koreanum* (Eko), and *E. pubescens* (Epu) using a MDF algorithm. As shown in Figure 2, almost all dots (*x* = integer, *y* = decimal) corresponding to the flavone in the data library are distributed within the five-point frame, suggesting the reliable potential of the pentagon for compounds-of-interest filtering. The captured MS^1^ information in the region was further analyzed to identify the chemical compounds in Esa, Eko, and Epu after the assignments of MS^2^ spectra.

### 2.3. Mass Fragmentation Behaviors of Flavonoids

**Figure 3:**
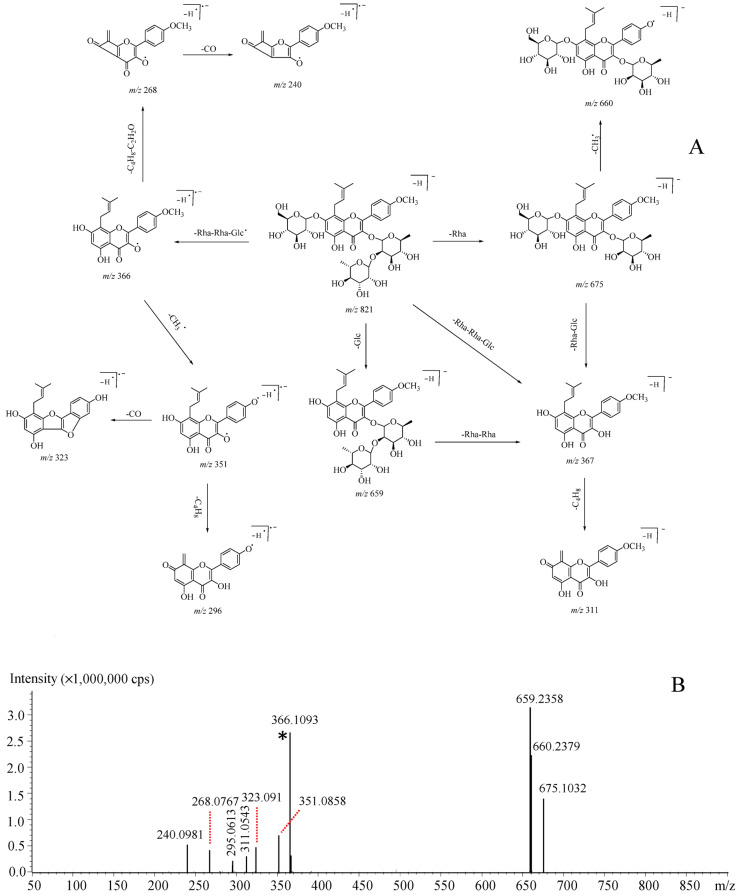
Proposed mass fragmentation pathways (**A**) and MS^2^ spectrum (**B**) of *m*/*z* 821 ([M–H]^−^) for epimedin C, a representative isopentenyl flavonoid in *Epimedium* plants. *: [Y_0_–H·]^−^.

As shown in Table S1 (Supporting Information), the flavonoids from *Epimedium* plants usually bear isopentenyl substitute(s) at the C-8 and/or C-6 site(s), and a large portion also contain glycosyl residues. Therefore, neutral losses of C_4_H_8_ (56 Da) and C_3_H_6_ (42 Da) usually occur, and neutral cleavages of glycosyl residues, such as glucose and rhamnose, are frequently observed. Here, epimedin C (**32**) that bears glucosyl, rhamnosyl, and isopentenyl substitution, was employed as a representative example to illustrate the mass fragmentation rules. The deprotonated molecular ion ([M–H]^−^) was observed at *m*/*z* 821. Multi-stage mass spectral signals were generated at *m*/*z* 675, 660, 659, 367, 366, 351, 323, 311, 295, 268, and 240. Fragment ion species at *m*/*z* 675 and 659 should be attributed to the neutral losses of rhamnosyl (C_6_O_4_H_10_, 146 Da) and glucosyl (C_6_O_5_H_10_, 162 Da) residues and the peak at *m*/*z* 660 should be generated by methyl radical cleavage (CH_3_·, 15 Da) from *m*/*z* 675. Noteworthily, both *m*/*z* 367 and 366 ions were observed and assigned as Y_0_^−^ and [Y_0_–H·]^−^, respectively, because of the glycosidation at C-3 of the aglycone [23,24,25]. Afterwards, signals at *m*/*z* 351, 323, 296, 268, and 240 should be generated by the methyl radical cleavage (CH_3_·, 15 Da), the methyl radical cleavage (CH_3_·, 15 Da) plus neutral cleavage of carbon monoxide (CO, 28 Da), the methyl radical cleavage (CH_3_·, 15 Da) plus neutral cleavage of C_4_H_8_ (56 Da), neutral losses of C_4_H_8_ (56 Da) and C_2_H_2_O(42 Da), and neutral losses of C_4_H_8_ (56 Da), C_2_H_2_O cleavage (42 Da) and carbon monoxide (CO, 28 Da), accordingly, from [Y_0_–H·]^−^ (*m*/*z* 366), whilst *m*/*z* 311 was yielded by C_4_H_8_ cleavage (56 Da) from Y_0_^−^(*m*/*z* 367). The proposed mass fragmentation pathways responsible for the primary signals observed in MS^2^ spectrum are seen in Figure 3.

### 2.4. Chemical Characterization of Epimedium Plants

Both negative and positive ionization polarities were utilized for mass spectral acquisition. In the positive mode, mainly [M+H]^+^ ions usually occupied the dominant role in MS^1^ spectra, and nonetheless, less fragment ion species were observed in the tandem mass spectra. On the other hand, in negative mode, fruitful fragment ion species were generated when [M–H]^−^ or [M+HCOO]^−^ ions entered the collision chamber. Therefore, [M–H]^−^ or [M+HCOO]^−^ ions could be introduced for the MDF frame to capture flavonoids, and moreover, those fragment ion species were able to suggest substructures. Base peak chromatograms of *E. sagittatum* (ESa, A), *E. koreanum* (EKo, B) and *E. pubescens* (EPu, C) that were recorded with negative ionization polarity are illustrated in Figure 1, and overall, great similarity occurs, obviously, among the chemical profiles of these three species.

Regarding prenylated flavonoid glycosides from *Epimedium* plants, four different skeletons have been reported (Table S1) on the basis of substituent groups, such as hydrogen, hydroxyl and methoxyl groups at the C–3′ and C–4′ sites of the B–ring. These flavonoids were defined as skeleton A (Y_0_^−^ at *m*/*z* 353), such as ikarisoside C (**12**), skeleton B (Y_0_^−^ at *m*/*z* 367), such as icariin (**35**), skeleton C (Y_0_^−^ at *m*/*z* 383), such as caohuoside C (**65**), as well as skeleton D (Y_0_^−^ at *m*/*z* 369), such as 3-*O*-rhamnosyl-3′,4′-hydroxyicariine (**53**), respectively. Regarding the glycosylation pattern, 3-*O*-, 7-*O*-, or 3,7-*di*-*O*-glycosyl substitutions were observed, and the glycosyl substitutes included glucosyl, rhamnosyl and xylosyl groups. It is worthwhile to mention that 3-*O*- or 7-*O*-saccharide substitution significantly affected the mass fragmentation behaviors. Based on the mass fragmentation rules summarized from the known standards, the structures of unknown constituents were tentatively characterized, and particularly, the identities of a total of 5 compounds were consolidated with authentic compounds. The retention time, accurate mass of deprotonated molecular ions and fragment ions for all putative identities, 99 in total, are summarized in Table 1. Except for compounds **4**–**5**, **11**, **20**, **22**, **25**, **28**, **36**–**37**, **41**, **45**–**46**, **51**–**53**, **57**–**60**, **66**, **68**–**69**, **71**, **73**, **75**–**76**, **83**, **89**, **92**–**93**, **95**, **98**–**99**, another 66 ones were detected in Esa, and on the other hand, **84** (minus compounds **7**, **10**, **13**, **17**, **25**, **40**, **42**, **51**, **66**, **67**, **85**, **91**, **94**, and **98**–**99**) and 66 flavonoids (minus compounds **4**–**6**, **11**, **20**, **22**, **28**–**29**, **36**, **40**–**42**, **44**–**46**, **52**–**53**, **57**–**60**, **67**–**69**, **71**, **73**, **76**, **83**, **85**, **89**, **92**–**93**, and **95**) occurred in Eka and Epu, respectively. Interestingly, some diagnostic compounds could be defined for each species, such as compound **36** for Eko, compound **25** for Epu, and compound **85** for Esa, because of their unique distribution patterns, and their potential for plant identity authentication, desirable for more validation assays.

**Figure 4:**
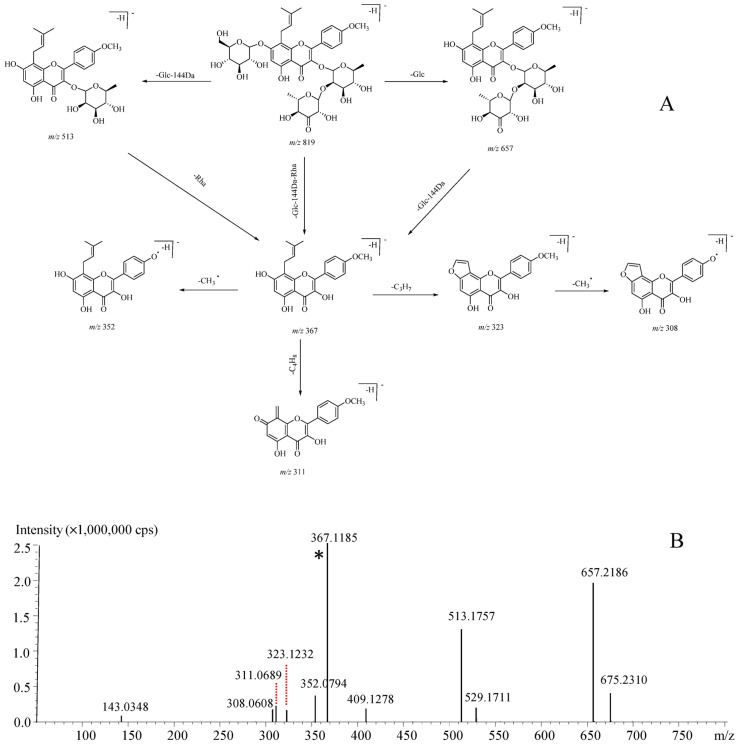
Proposed mass cracking pathways (**A**) and MS^2^ spectrum (**B**) for oxidized epimedin C that was featured by the 144 Da neutral loss. *: Y_0_^−^.

Noteworthily, neutral loss of 144 Da was firstly assigned to the cleavage of deprotonated rhamnosyl residue [26] and this phenomenon was observed for each *Epimedium* species. Compound **49** for instance, whose molecular formula was calculated as C_39_H_48_O_19_, generated a [M–H]^−^ ion at *m*/*z* 819. Firstly, the [M–H]^−^ ion produced a prominent ion at *m*/*z* 367 in the MS^2^ spectrum, suggesting a neutral loss of a 7-*O*-glucosyl residue (162 Da) and a 3-*O*-rhamnosyl-deprotonated rhamnosyl residue (290 Da). Afterwards, the Y_0_^−^ ion was automatically selected for further dissociation to generate a moderate strength ion at *m*/*z* 352, indicating a methyl radical cleavage. Moreover, a primary fragment ion at *m*/*z* 311 was also detected, which was assigned as the cleavage of C_4_H_8_ (56 Da) from the isopentenyl at the C-8 position. Hence, this compound was identified as oxidized epimedin C. The proposed mass fragmentation pathways are shown in Figure 4A and the primary signals in the MS^2^ spectrum and shown in Figure 4B. Compared to oxidized epimedin C, epimedin C gave a deprotonated molecular ion ([M–H]^−^) at *m*/*z* 821 as the base peak. It is speculated that a deprotonated rhamnosyl residue accounts for the mass difference.

In addition to the aforementioned compounds, there are a set of acetyl–substituted isopentenyl flavonoids in *Epimedium* plants, and this acetylation usually occurs on the glycosyl groups, such as monoacetylated glucosyls (**28**, neutral loss of 204 Da), monoacetylated rhamnosyls (**94**, neutral loss of 188 Da), monoacetylated xylosyls (**60**, neutral loss of 174 Da), diacetylated glucosyls (**69**, neutral loss of 246 Da) and so on. Similarly, all peaks were tentatively or unambiguously identified and their MS data are shown in Table 1.

## 3. Experimental

### 3.1. Materials and Chemicals

Authentic compounds, including epimedins A–C, icariin, and baohuoside I, were commercially supplied by Yuanye Bio–Technology Co., Ltd. (Shanghai, China), Ruifensi Bio–Technology Co., Ltd. (Chengdu, China), and Standard Bio–Technology Co., Ltd. (Shanghai, China). The purity of each reference compound was determined to be greater than 99% by LC-UV–MS.

LC–MS grade methanol, acetonitrile (ACN), and formic acid were purchased from Thermo–Fisher (Pittsburgh, PA, USA). Deionized water was prepared in–house using a Milli–Q integral water purification system (Millipore, Bedford, MA, USA). The other chemicals were of analytical grade and commercially supplied by Beijing Chemical Works (Beijing, China).

**Figure 5:**
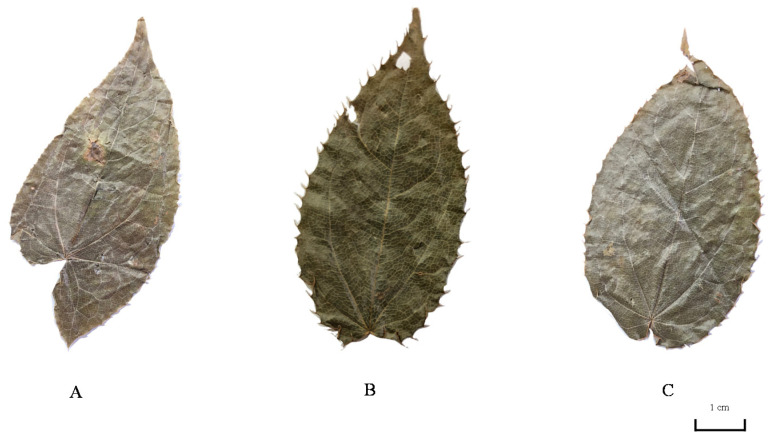
The dried leaves of ESa (**A**), Eko (**B**) and EPu (**C**).

Three batches of raw materials that were identified as Epimedii Herba were supplied by Guizhou Hanfang Pharmaceutical Co., Ltd. (Guiyang, China). Their original sources were authenticated as the dried leaves of *Epimedium sagittatum* (Sieb. *et* Zucc.) Maxim., *E. koreanum* Nakai., and *E. pubescens* Maxim. (Figure 5), Esa, Eko, and Epu in short, respectively, according to their microscopic and macroscopic features by Prof. Yuan Zhang from Beijing University of Chinese Medicine. All voucher specimens are deposited in the herbarium of Modern Research Center for Traditional Chinese Medicine, School of Chinese Materia Medica, Beijing University of Chinese Medicine (Beijing, China).

### 3.2. Preparation of Extraction Vessel

After being thoroughly dried in an oven (0 °C, 3 days), all raw materials were pulverized into powders using a sample mill (model YF102, Yongli Pharmacy Machinery Company, Ruian, Zhejiang, China), and then individually sieved through a 50-mesh (0.25 mm, I.D.) grid. A 4 mg aliquot of powder was sampled from each batch, and completely dispersed using 10 mg diatomaceous earth that was previously washed with 5.0 mL 50% aqueous methanol. The mixture was totally transferred into a hollow guard column (3.0 × 4.0 mm i.d., Phenomenex) that was afterwards sealed with two filter membranes (0.22 μm) and two caps to yield the extraction cell. Then, the filled guard column was placed into an adapted Phenomenex Security Guard™ (Torrance, CA, USA) cartridge holder to generate the extraction vessel.

### 3.3. OLE–LC–DAD–IT-TOF-MS Measurement

**Figure 6:**
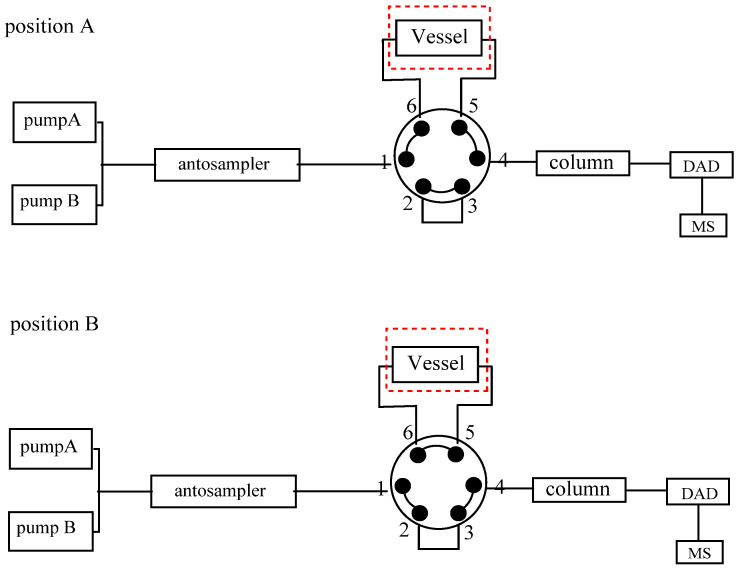
The schematic of OLE–LC–DAD–IT–TOF–MS. Each measurement is divided into two phases, including extraction (0–5 min) and elution (5–42 min) phases via switching the electronic valve from position I to position II.

The OLE–LC–DAD–IT-TOF-MS instrumentation (Figure 6) was configured by referring to the schematic described in our previous article [12]. Minor modifications were performed by removing both the dilution pumps and HILIC column. All building blocks were Shimadzu products (Kyoto, Japan). The prepared extraction vessel was inserted into the integrated system and maintained in the thermal column oven (75 °C). Each OLE–LC–IT-TOF-MS measurement, 42 min in total, was divided into extraction (0–5 min, Figure 6, position A) and elution (5–42 min, Figure 6, position B) phases by automatically switching the electronic 6-port/2-position valve. The entire extraction phase was maintained for 5 min, a steel tube (650 × 0.13 I.D. mm) was introduced connect the vessel and the column, was maintained in the column oven to efficiently warm the solvent and crude materials. After the extraction was completed, the electronic 6-port/2-position valve was automatically switched to position B, corresponding to the on-line elution phase. Chromatographic separations were conducted on a Waters Acquity UPLC HSS T3 column, that was maintained at atmosphere temperature (23 °C). The mobile phase was composed by 0.1% aqueous formic acid (A) and acetonitrile (B). Regarding extraction phase, the valve was maintained at position A, and 10% B was delivered at a flow rate of 0.3 mL/min to transmit those compounds from plant cells onto the head of the analytical column. Pressurized warm water extraction was then achieved because of the back–pressure (approximately 30 MPa) of the column. The valve was afterwards transferred to position B, corresponding to the elution phase, and the gradient elution of the column at a flow rate of 0.2 mL/min for another 37 min was programmed as follows: 5–5.1 min, 10–25% B; 5.1–13 min, 25–27% B; 13–29 min, 27–48% B; 29–32 min, 48–80% B; 32–35 min, 80–100% B; 35–35.1 min, 100–10% B; and 35.1–42 min, 10% B. UV length was defined at full wavelength (190–400 nm) for signal detection.

An IT-TOF-MS system (Shimadzu, Kyoto, Japan) was in charge of monitoring the column effluent and an electrospray ionization (ESI) interface was connected to the column outlet. Both positive and negative ionization polarities were applied and the primary parameters were defined as below: nebulizing gas flow rate, 1.5 L/min; drying gas pressure, 100 MPa; detector voltage, 1.40 kV; curved desolvation line (CDL) temperature, 200 °C; block heater temperature, 200 °C; interface voltage, 1.6 kV; and IT vacuum, 1.9 × 10^−2^ Pa. Full MS^1^ scan (*m*/*z* 100–1000) and automated multiple stage scan (*m*/*z* 50–1000) were applied for spectral acquisition. The ion accumulation time was set at 100 ms, and the collision energy level was defined as 45% for collision induced dissociation (CID).

### 3.4. In–House Chemical Library Construction

As one of the most favored herbal medicines, chemical characterization and phytochemical isolation have been widely conducted for *Epimedium* plants. In the current study, extensive attention was paid to information collection from available internet sources, such as PubMed, ChemSpider, Reaxys, Scifinder, CNKI, Google Scholar, and Web of Science. As a result, approximately 277 flavonoids (Table S1) were identified in previous reports and included in the in-house chemical library. All annotated compounds were flavonoids, and several subtypes such as demethylanhydroicaritin glycoside, anhydroicaritin glycoside, 3′-hydroxyicariine glycoside, 3′,4′-dihydroxyicariine glycoside, simple prenylflavonoids, as well as other flavonoids, were involved. The structural and tandem mass spectral information is summarized in Table S1. All theoretical values of the quasi–molecular ions, usually [M–H]^−^, were divided into integer and decimal parts, and imported into Excel (Office2010, Microsoft, Redmond, WA, USA) to draw the pentagonal frame (Figure 2) for rapid information filtering. Moreover, the mass fragmentation pathways proposed in the literature were confirmed with the collected authentic compounds as well as the signals occurring in the current case, and were afterwards applied for structural annotation.

## 4. Discussion and Conclusions

The tedious sample preparation procedure not only represent a significant technical barrier for high-throughput measurements, but results in the fact that labile compounds risk exposure to organic solvents as well as some other environmental parameters. High temperature and pressure are able to accelerate the shuttling back and forth of the extraction solvent for the plant cells, thus advancing the extraction efficiency. In the current study, a column oven at 75 °C and the significant back-pressure (about 30 MPa) jointly promoted the extraction course. Moreover, those less polar flavonoids were also captured although the use of 10% aqueous ACN as extraction solvent, because the molecular distances increased with the temperature increment and the polarity was consequently decreased to enable the extraction of less polar compounds. Therefore, efficient and universal extraction was achieved with the OLE module. However, further efforts are desirable to improve the instrumentation to achieve automated measurements, because in its current status, only a single extraction vessel is permitted in the instrumental configuration.

Because of the rapid development of mass spectrometric techniques, the resolution and scan rates have been significantly improved and consequently, the dataset size has increased dramatically. As a consequence, it is urgent to pursue a fit-for-purpose approach to process the massive dataset, and MDF, neutral loss filtering (NLF) as well as diagnostic fragment ion filtering (DFIF) have been demonstrated as the best choices. Each one requires prior knowledge for the chemical family-of-interest, resulting in risk of missing information when the information isn’t comprehensively collected. Moreover, it is challenging for such approaches to accomplish universally chemical profiling because of the chemical diversity in a given HM. In the current study, we merely focused on the flavonoids, and fortunately, a mass of information is archived in the literature as well as accessible databases. The information composed of the in–house data library to summarize the mass defect rules of flavonoids, thus significantly accelerating information filtering with the pentagon frame. Actually, greater information missing risk usually results from NLF or DFIF, because these two approaches rely on multi–stage mass spectra (mainly MS^2^ spectra) and appropriate collision energy plays the determinant role for the MS^2^ spectra generation. Otherwise, a lot of information might be filtered when an inappropriate collision energy is applied for MS^2^ spectral acquisition. On the other hand, MDF merely concerns MS^1^ spectral information and therefore has greater potential for universal information capturing, however, this results in more redundant information. In further studies, molecular weight imprinting might be an appropriate way in the near future to accurately capture the desired information without the involvement of redundant information, through applying the molecular information for the components reported in the literature. Particularly, molecular weight imprinting technique might be extremely suitable for those well–studied HMs, e.g., Epimedii Herba, because its chemical profiles has been extensively studied and abundant prior knowledge is available.

In current study, to achieve direct LC–MS measurement and rapid post–acquisition data processing, OLE–LC–IT-TOF-MS coupled with a mass defect filtering approach was proposed and three original sources of Epimedii Herba were employed to illustrate and validate the strategy applicability. Rapid MS^1^ spectral information filtering was achieved by MDF. A total of 99 flavonoids were characterized from Epimedii Herba based on their mass spectra. Noteworthily, neutral loss of 144 Da was firstly assigned to the cleavage of deprotonated rhamnosyl residue. Significant species–differences didn’t occur among their chemical profiles of the three *Epimedium* plants. The current study proposes a robust strategy enabling rapid chemical profiling of, but not limited to, HMs.

## Figures and Tables

**Table 1:** Mass spectral data and plausible identities of the chemical compounds from *Epimedium* plants by OLE–UHPLC–IT-TOF-MS.

No.	*t*_R_ (min)	Molecular Formula	[M–H]^−^	Error (ppm)	MS/MS	Esa	Eko	Epu	Putative Identity
1	8.665	C_21_H_20_O_13_	479.0822	−1.88	317.0082[M–H–Glc]^−^	√	√	√	myricetin-3-O-glc
2	9.242	C_21_H_20_O_13_	463.0867	−3.23	301.0200[M–H–Glc]^−^;283.0111[M–H–Glc–H_2_O]^−^;255.0173[M–H–Glc–CO–H_2_O]^−^	√	√	√	quercetin-3-O-gal
3	9.283	C_21_H_20_O_13_	463.0872	−2.16	301.9988[M–H–Glc]^−^;273.0003[M–H–Glc–CO]^−^;254.9923[M–H–Glc–CO–H_2_O]^−^	√	√	√	quercetin-7-O-glc
4	9.353	C_32_H_40_O_16_	679.2232	−1.77	517.1674[M–H–Glc]^−^;355.1254[M–H–2Glc]^−^;337.1009[M–H–2Glc–H_2_O]^−^;327.1169[M–H–2Glc–CO]^−^;311.1223[M–H–2Glc–CO_2_]^−^;283.1262[M–H–2Glc–CO–CO_2_]^−^;219.0596[^1,3^A]^−^	-	√	-	dihydrodemethylicaritin-7-O-glc-glc
5	9.658	C_32_H_38_O_16_	677.2095	1.18	515.1480[M–H–Glc]^−^;369.0903[M–H–2Glc–Rha]^−^;219.0586[^1,3^A]^−^	-	√	-	3′,4′-hydroxyicariine-3-O-rha-glc-7-O-glc
6	9.765	C_32_H_38_O_16_	677.2096	1.33	515.1478[M–H–Glc]^−^;353.0952[M–H–2Glc]^−^;323.0838[M–H–2Glc–CHO]^−^;297.0987[M–H–2Glc–CHO–C_4_H_8_]^−^	√	√	-	demethylanhydroicaritin-3-O-glc-7-O-glc
7	10.182	C_26_H_28_O_15_	579.1359	0.69	301.1319[M–H–Glc–Xyl]^−^;283.0337[M–H–Glc–Xyl–H_2_O]^−^;255.0192[M–H–Glc–Xyl–H_2_O–CO]^−^;151.0047[^1,3^A]^−^	√	-	√	quercetin-3-O-rha-xyl
8	10.640	C_38_H_48_O_19_	807.2696	−2.60	661.2279[M–H–Rha]^−^;645.2201[M–H–Glc]^−^;499.1581[M–H–Rha–Glc]^−^;353.0942[M–H–2Rha–Glc]^−^;323.0910[M–H–2Rha–Glc–CHO]^−^;297.0972[M–H–2Rha–Glc–C_4_H_8_]^−^	√	√	√	demethylanhydroicaritin-3-O-rha-rha-7-O-glc
9	10.747	C_21_H_20_O_11_	447.0934	0.22	285.0337[M–H–Glc]^−^;257.0192[M–H–Glc–CO]^−^	√	√	√	kaempferol-7-O-glc
10	10.865	C_27_H_30_O_15_	593.1499	−2.19	447.0937[M–H–Rha]^−^;301.0341[M–H–2Rha]^−^	√	-	√	quercetin-3-O-rha-7-O-rha
11	10.918	C_21_H_20_O_7_	383.1168	8.35	237.0686[^1,3^A+H_2_O]^−^;219.0578[^1,3^A]^−^	-	√	-	aglycone
12	10.950	C_38_H_48_O_20_	823.2679	1.58	661.2143[M–H–Glc]^−^;515.1463[M–H–Glc–Rha]^−^;353.1012[M–H–2Glc–Rha]^−^;297.09658[M–H–2Glc–Rha–C_4_H_8_]^−^	√	√	√	ikarisoside C
13	11.313	C_37_H_46_O_19_	793.2564	0.38	631.1978[M–H–Glc]^−^;499.1496[M–H–Glc–Xyl]^−^;353.0946[M–H–Glc–Xyl–Rha]^−^;281.0386[M–H–Glc–Xyl–Rha–C_5_H_12_]^−^	√	-	√	epimedoside E
14	11.463	C_38_H_48_O_19_	807.2707	−1.24	645.2193[M–H–Glc]^−^;499.1482[M–H–Glc–Rha]^−^;353.1007[M–H–Glc–2Rha]^−^;281.0442[M–H–Glc–2Rha–C_5_H_12_]^−^;219.0506[^1,3^A]^−^	√	√	√	demethylanhydroicaritin-3-O-rha(1-3)-rha-7-O-glc
15	11.538	C_32_H_38_O_15_	661.2146	1.21	515.1517[M–H–Glc]^−^;353.1037[M–H–Glc–Rha]^−^;323.0909[M–H–Glc–Rha–CHO]^−^	√	√	√	icarisoside B
16	11.998	C_27_H_30_O_14_	577.1564	0.17	431.0907[M–H–Rha]^−^;285.0330[M–H–2Rha]^−^;255.0225[M–H–2Rha–CH_2_O]^−^	√	√	√	kaempferol-3-O-rha-7-O-rha
17	12.158	C_26_H_28_O_14_	563.1407	0.18	431.0899[M–H–Xyl]^−^;285.0301[M–H–Xyl–Rha]^−^;255.0258[M–H–Xyl–Rha–CH_2_O]^−^	√	-	√	kaempferol-7-O-rha-xyl
18	13.257	C_21_H_20_O_10_	431.0987	0.70	285.0394[M–H–Rha]^−^;255.0339[M–H–Rha–CH_2_O]^−^;227.0342[M–H–Rha–CH_2_O–CO]^−^;151.0028[^1,3^A]^−^	√	√	√	kaempferol-3-O-rha
19	13.707	C_39_H_50_O_20_	837.2842	2.27	675.2303[M–H–Glc]^−^;383.1121[M–H–Glc–2Rha]^−^;353.1037[M–H–Glc–2Rha–CH_2_O]^−^	√	√	√	3′-hydroxyicariine-3-O-rha-rha-7-O-glc
20	14.038	C_26_H_30_O_11_	517.1719	0.77	355.1273[M–H–Glc]^−^;327.1228[M–H–Glc–CO]^−^;311.1286[M–H–Glc–CO_2_]^−^;283.1331[M–H–Glc–CO–CO_2_]^−^	-	√	-	dihydrodemethylicaritin-7-O-glc
21	14.070	C_33_H_40_O_16_	691.2255	1.59	545.1596[M–H–Rha]^−^;529.1649[M–H–Glc]^−^;383.1059[M–H–Glc–Rha]^−^	√	√	√	3′-hydroxyicariine-3-O-rha-7-O-glc
* 22	14.770	C_39_H_48_O_20_	835.2676	1.20	691.2228[M–H–C_6_H_8_O_4_]^−^;545.1645[M–H–C_6_H_8_O_4_–Rha]^−^;383.1126[M–H–C_6_H_8_O_4_–Rha–Glc]^−^;312.0547[M–H–C_6_H_8_O_4_–Rha–Glc–C_4_H_8_–CH_3_·]^−^	-	√	-	3′-hydroxyicariine-3-O- rha-C_6_H_8_O_4_-7-O-glc
* 23	14.958	C_38_H_46_O_19_	805.2588	3.35	661.2142[M–H–C_6_H_8_O_4_]^−^;515.1533[M–H–C_6_H_8_O_4_–Rha]^−^;353.1028[M–H–C_6_H_8_O_4_–Rha–Glc]^−^	√	√	√	demethylanhydroicaritin-3-O-rha-glc-C_6_H_8_O_4_
24	16.065	C_39_H_50_O_20_	837.2836	1.56	675.2288[M–H–Glc]^−^;529.1688[M–H–Glc–Rha]^−^;367.1177[M–H–2Glc–Rha]^−^;352.0950[M–H–2Glc–Rha–CH_3_·]^−^	√	√	√	hexandroside F
25	16.958	C_42_H_52_O_22_	907.2888	1.21	745.2351[M–H–Glc]^−^;703.2230[M–H–Glc(OAc)]^−^;515.1547[M–H–Glc(OAc)–Rha(OAc)]^−^;353.1024[M–H–Glc(OAc)–Rha(OAc)–Glc]^−^	-	-	√	demethylanhydroicaritin-3-O-rha(OAc)-glc(OAc)-7-O-glc
^a^ 26	17.142	C_39_H_50_O_20_	837.2849	3.10	675.2307[M–H–Glc]^−^;367.1169[M–H–2Glc–Rha]^−^;352.0932[M–H–2Glc–Rha–CH_3_·]^−^;323.0905[M–H–2Glc–Rha–CH_3_·–CHO]^−^;311.0542[M–H–2Glc–Rha–C_4_H_8_]^−^	√	√	√	epimedium A
27	17.335	C_33_H_40_O_15_	675.2302	1.18	513.1752[M–H–Glc]^−^;367.1185[M–H–Glc–Rha]^−^;352.0942[M–H–Glc–Rha–CH_3_·]^−^;323.0921[M–H–Glc–Rha–CH_3_·–CHO]^−^;295.0614[M–H–Glc–Rha–CH_3_·–CHO–CO]^−^;279.1027[M–H–Glc–Rha–CH_3_·–CHO–CO–H_2_O]^−^	√	√	√	icariin isomer
28	17.752	C_40_H_50_O_21_	865.2784	1.39	703.2242[M–H–Glc]^−^;661.2138[M–H–Glc(OAc)]^−^;353.0939[M–H–Glc(OAc)–Glc–Rha]^−^;323.0908[M–H–Glc(OAc)–Glc–Rha–CHO·]^−^	-	√	-	demethylanhydroicaritin-3-O-rha-glc(OAc)-7-O-glc
29	17.848	C_38_H_48_O_19_	807.2736	2.35	645.2190[M–H–Glc]^−^;366.1096[M–H–Glc–Rha–Xyl–H·]·^−^;351.0857[M–H–Glc–Rha–Xyl–CH_3_·–H·]·^−^;323.0898[M–H–Glc–Rha–Xyl–CH_3_·–CO–H·]·^−^;311.0558[M–H–Glc–Rha–Xyl–C_4_H_8_]^−^;295.0609[M–H–Glc–Rha–Xyl–CH_3_·–2CO–H·]·^−^;279.1021[M–H–Glc–Rha–Xyl–CH_3_·–2CO–H_2_O–H·]·^−^;219.0509[^1,3^A]^−^	√	√	-	epimedin B isomer
30	17.998	C_32_H_38_O_14_	645.219	0.15	513.1758[M–H–Xyl]^−^;367.1173[M–H–Xyl–Rha]^−^;352.0929[M–H–Xyl–Rha–CH_3_·]^−^;323.0917[M–H–Xyl–Rha–CH_3_·–CHO]^−^;311.0557[M–H–Xyl–Rha–C_4_H_8_]^−^;295.0611[M–H–Xyl–Rha–CH_3_·–CHO–CO]^−^;279.1023[M–H–Xyl–Rha–CH_3_·–CHO–CO–H_2_O]^−^	√	√	√	sagittatoside B isomer
^a^ 31	18.052	C_38_H_48_O_19_	807.2734	−4.70	645.2189[M–H–Glc]^−^;367.1151[M–H–Glc–Rha–Xyl]^−^;351.0858[M–H–Glc–Rha–Xyl–CH_3_·]^−^;323.0907[M–H–Glc–Rha–Xyl–CH_3_·–CHO]^−^;311.0539[M–H–Glc–Rha–Xyl–C_4_H_8_]^−^;295.0607[M–H–Glc–Rha–Xyl–CH_3_·–CHO–CO]^−^;279.1031[M–H–Glc–Rha–Xyl–CH_3_·–CHO–CO]^−^	√	√	√	epimedin B
^a^ 32	18.293	C_39_H_50_O_19_	821.2897	−2.19	675.1032[M–H–Rha]^−^;659.2358[M–H–Glc]^−^;366.1093[M–H–Glc–2Rha–H·]·^−^;351.0858[M–H–Glc–2Rha–CH_3_·–H·]·^−^;323.0910[M–H–Glc–2Rha–CH_3_·–CO–H·]·^−^;311.0543[M–H–Glc–2Rha–C_4_H_8_]^−^;295.0613[M–H–Glc–2Rha–CH_3_·–2CO–H·]·^−^;268.0767[M–H–Glc–2Rha–CH_3_·–C_4_H_8_–C_2_H_2_O–H·]·^−^;240.0981[M–H–Glc–2Rha–CH_3_·–C_4_H_8_–C_2_H_2_O–CO–H·]·^−^	√	√	√	epimedium C
33	18.405	C_39_H_50_O_19_	821.2874)	−6.08	659.2367[M–H–Glc]^−^;366.1097[M–H–Glc–2Rha–H·]·^−^;351.0857[M–H–Glc–2Rha–CH_3_·–H·]·^−^;323.0905[M–H–Glc–2Rha–CH_3_·–CO–H·]·^−^;311.0553[M–H–Glc–2Rha–C_4_H_8_]^−^;295.0606[M–H–Glc–2Rha–CH_3_·–2CO–H·]·^−^;279.1015[M–H–Glc–2Rha–CH_3_·–2CO–H_2_O–H·]·^−^	√	√	√	hexandroside D
* 34	18.982	C_39_H_48_O_19_	819.2725	0.98	657.2186[M–H–Glc]^−^;513.1760[M–H–Glc–C_6_H_8_O_4_]^−^;367.1174[M–H–Glc–C_6_H_8_O_4_–Rha]^−^;323.0893[M–H–Glc–C_6_H_8_O_4_–Rha–CH_3_·–CO]^−^	√	√	√	anhydroicaritin-3-O-rha-C_6_H_8_O_4_-7-O-glc isomer
^a^ 35	19.187	C_33_H_40_O_15_	721.2378	4.02	529.1713[M–H–Rha]^−^;513.1766[M–H–Glc]^−^;366.1077[M–H–Glc–Rha–H·]·^−^;351.0839[M–H–Glc–Rha–CH_3_·–H·]·^−^;323.0569[M–H–Glc–Rha·–CH_3_·–CO–H·]·^−^;311.0534[M–H–Glc–Rha–C_4_H_8_]^−^	√	√	√	icariin
36	19.282	C_41_H_52_O_21_	879.2958	3.41	717.2405[M–H–Glc]^−^;675.2573[M–H–Glc–OAc]^−^;513.1764[M–H–2Glc–OAc]^−^;367.1182[M–H–2Glc–Rha(OAc)]^−^;349.1131[M–H–2Glc–Rha(OAc)–H_2_O]^−^	-	√	-	anhydroicaritin-3-O-rha(1-4OAc)-(1-3)glc-7-O-glc
37	19.528	C_39_H_46_O_18_	801.2631	2.5	639.2079[M–H–Glc]^−^;513.1766[M–H–Glc–3OAc]^−^;367.1184[M–H–Glc–Rha(3OAc)]^−^;352.0862[M–H–Glc–Rha(3OAc)–CH_3_·]^−^;323.0907[M–H–Glc–Rha(3OAc)–CH_3_·–CHO]^−^	-	√	√	anhydroicaritin-3-O- glc-7-O-rha-3OAc
* 38	19.795	C_39_H_48_O_19_	819.2738	2.56	657.2188[M–H–Glc]^−^;513.1760[M–H–Glc–C_6_H_8_O_4_]^−^;367.1178[M–H–Glc–C_6_H_8_O_4_–Rha]^−^;352.0920[M–H–Glc–C_6_H_8_O_4_–Rha–CH_3_·]^−^	√	√	√	anhydroicaritin-3-O-rha-C_6_H_8_O_4_-7-O-glc
* 39	19.848	C_39_H_48_O_19_	865.2804	3.7	657.2187[M–H–Glc]^−^;513.1763[M–H–Glc–C_6_H_8_O_4_]^−^;367.1183[M–H–Glc–C_6_H_8_O_4_–Rha]^−^;352.0933[M–H–Glc–C_6_H_8_O_4_–Rha–CH_3_·]^−^;323.0902[M–H–Glc–C_6_H_8_O_4_–Rha–CH_3_·–CHO]^−^	√	√	√	anhydroicaritin-3-O-rha-7-O-rha-C_6_H_8_O_4_
* 40	20.105	C_39_H_48_O_21_	851.264	2.94	689.2095[M–H–Glc]^−^;513.1757[M–H–Glc–C_6_H_8_O_6_]^−^;367.1178[M–H–Glc–C_6_H_8_O_6_–Rha]^−^	√	-	-	anhydroicaritin-3-O-rha-C_6_H_8_O_6_-7-O-glc
41	20.672	C_44_H_54_O_23_	949.3009	2.74	787.2455[M–H–Glc]^−^;745.2338[M–H–Glc(OAc)]^−^;515.1537[M–H–Glc(OAc)–Rha(2OAc)]^−^;353.1026[M–H–Glc(OAc)–Rha(2OAc)–Glc]^−^	-	√	-	demethylanhydroicaritin-3-O-rha(2OAc)-glc(OAc)-7-O-glc
42	20.722	C_32_H_38_O_16_	677.2045	−6.2	515.1427[M–H–Glc]^−^;369.0838[M–H–Glc–Rha]^−^	√	-	-	3′,4′-hydroxyicariine-7-O-rha-glc
* 43	20.758	C_39_H_48_O_19_	865.2811	4.51	657.2185[M–H–Glc]^−^;513.1768[M–H–Glc–C_6_H_8_O_4_]^−^;367.1188[M–H–Glc–C_6_H_8_O_4_–Rha]^−^	√	√	√	anhydroicaritin-3-O-rha-7-O-glc-C_6_H_8_O_4_
* 44	20.768	C_39_H_48_O_21_	851.2637	2.58	689.2068[M–H–Glc]^−^;513.1756[M–H–Glc–C_6_H_8_O_6_]^−^;367.1175[M–H–Glc–C_6_H_8_O_6_–Rha]^−^	√	√	-	anhydroicaritin-3-O-glc-7-O-rha-C_6_H_8_O_6_
45	20.875	C_35_H_42_O_16_	717.2399	−0.14	513.1752[M–H–Glc(OAc)]^−^;367.1176[M–H–Glc(OAc)–Rha]^−^;352.0943[M–H–Glc(OAc)–Rha–CH_3_·]^−^;323.0902[M–H–Glc(OAc)–Rha–CH_3_·–CO]^−^;311.0559[M–H–Glc(OAc)–Rha–C_4_H_8_]^−^	-	√	-	anhydroicaritin-3-O-glc(OAc)-7-o-rha
46	20.928	C_44_H_54_O_23_	949.3003	2.11	787.2454[M–H–Glc]^−^;745.2341[M–H–Glc(OAc)]^−^;515.1541[M–H–Glc(OAc)–Rha(2OAc)]^−^;353.1022[M–H–Glc(OAc)–Rha(2OAc)–Glc]^−^	-	√	-	demethylanhydroicaritin-3-O-rha(2OAc)-glc(OAc)-7-O-glc
47	21.035	C_39_H_50_O_19_	821.2846	−3.4	659.1975[M–H–Glc]^−^;367.1174[M–H–Glc–Rha–Rha]^−^	√	√	√	epimedium C isomer
48	21.228	C_39_H_48_O_20_	835.2709	5.15	673.2146[M–H–Glc]^−^;529.1706[M–H–Glc–C_6_H_8_O_4_]^−^;367.1191[M–H–2Glc–C_6_H_8_O_4_]^−^;352.0939[M–H–2Glc–C_6_H_8_O_4_–CH_3_·]^−^	√	√	√	anhydroicaritin-3-O- glc-C_6_H_9_O_4_-glc
* 49	21.400	C_39_H_48_O_19_	819.2755	4.64	657.2186[M–H–Glc]^−^;513.1757[M–H–Glc–C_6_H_8_O_4_]^−^;367.1185[M–H–Glc–C_6_H_8_O_4_–Rha]^−^;352.0794[M–H–Glc–C_6_H_8_O_4_–Rha–CH_3_·]^−^;323.1232[M–H–Glc–C_6_H_8_O_4_–Rha–C_3_H_7_]^−^;311.0689[M–H–Glc–C_6_H_8_O_4_–Rha–C_4_H_8_]^−^;308.0608[M–H–Glc–C_6_H_8_O_4_–Rha–C_3_H_7_–CH_3_·]^−^	√	√	√	anhydroicaritin-3-O-rha-C_6_H_8_O_4_-7-O-glc
* 50	21.853	C_39_H_48_O_19_	819.2741	2.93	657.2186[M–H–Glc]^−^;513.1758[M–H–Glc–C_6_H_8_O_4_]^−^;367.1185[M–H–Glc–C_6_H_8_O_4_–Rha]^−^	√	√	√	anhydroicaritin-3-O-glc-7-O-rha-C_6_H_8_O_4_
51	21.945	C_33_H_40_O_16_	691.2246	0.29	545.1596[M–H–Rha]^−^;529.1678[M–H–Glc]^−^;383.1042[M–H–Glc–Rha]^−^	-	-	√	3′-hydroxyicariine-3-O-glc-7-O-rha
52	22.318	C_43_H_54_O_22_	921.3065)	3.36	759.2501[M–H–Glc]^−^;717.2369[M–H–Glc(OAc)]^−^;367.1175[M–H–Glc(OAc)–Rha(OAc)–Glc]^−^;352.0932[M–H–Glc(OAc)–Rha(OAc)–Glc–CH_3_·]^−^;323.0888[M–H–Glc(OAc)–Rha(OAc)–Glc–CH_3_·–CHO]^−^	-	√	-	epimedokoreanoside I
53	22.565	C_26_H_28_O_11_	515.1556	−0.58	369.0957[M–H–Rha]^−^;219.0661[^1,3^A]^−^	-	√	-	3′,4′-hydroxyicariine-3-O-rha
54	22.942	C_32_H_38_O_15_	661.2143	0.76	499.1478[M–H–Glc]^−^;353.1006[M–H–Glc–Rha]^−^;323.0909[M–H–Glc–Rha–CHO]^−^;297.0409[M–H–Glc–Rha–C_4_H_8_]^−^;281.0452[M–H–Glc–Rha–C_5_H_12_]^−^;255.0299[M–H–Glc–Rha–C_5_H_12_–CH_2_CO]^−^	√	√	√	icarisoside B isomer
55	23.198	C_32_H_38_O_15_	661.2163	3.78	529.1716[M–H–Xyl]^−^;383.1125[M–H–Xyl–Rha]^−^;313.0654[[M–H–Xyl–Rha–C_4_H_7_–CH_3_·]^−^;179.0703[^0,3^B]^−^	√	√	√	3′-hydroxyicariine-3-O-rha-xyl
56	23.883	C_26_H_28_O_11_	515.1561	0.39	353.0991[M–H–Glc]^−^;323.0906[M–H–Glc–CHO]^−^;297.0969[M–H–Glc–Rha–C_4_H_8_]^−^	√	√	√	demethylanhydroicaritin-7-O-glc
57	24.155	C_43_H_54_O_22_	921.3052	1.95	759.2502[M–H–Glc]^−^;717.2382[M–H–Glc(OAc)]^−^;529.1704[M–H–Glc(OAc)–Rha(OAc)]^−^;367.1176[M–H–Glc(OAc)–Rha(OAc)–Glc]^−^;352.0936[M–H–Glc(OAc)–Rha(OAc)–Glc–CH_3_·]^−^;323.0905[M–H–Glc(OAc)–Rha(OAc)–Glc–CH_3_·–CHO]^−^	-	√	-	epimedokoreanoside I isomer
58	24.326	C_40_H_50_O_20_	849.2837	1.65	687.2276[M–H–Glc]^−^;555.2006[M–H–Glc–Xyl]^−^;513.1631[M–H–Glc–Xyl–OAc]^−^;367.1170[M–H–Glc–Xyl–Rha(OAc)]^−^	-	√	-	anhydroicaritin-3-O-rha(OAc)-xyl-7-O-glc
59	24.508	C_43_H_54_O_22_	921.3049	1.62	759.2512[M–H–Glc]^−^;367.1176[M–H–Glc(OAc)–Rha(OAc)–Glc]^−^;352.0945[M–H–Glc(OAc)–Rha(OAc)–Glc–CH_3_·]^−^;323.0888[M–H–Glc(OAc)–Rha(OAc)–Glc–CH_3_·–CHO]^−^	-	√	-	epimedokoreanoside I isomer
60	24.572	C_40_H_50_O_20_	849.2841	2.12	687.2276[M–H–Glc]^−^;513.0634[M–H–Glc–Xyl–OAc]^−^;367.1120[M–H–Glc–Xyl–Rha(OAc)]^−^	-	√	-	anhydroicaritin-3-O-rha-7-O-glc-xyl(OAc)
61	24.685	C_31_H_36_O_14_	631.2044	1.90	499.1482[M–H–Xyl]^−^;353.0961[M–H–Xyl–Rha]^−^;323.0914[M–H–Xyl–Rha–CHO·]^−^;281.0453[M–H–Xyl–Rha–C_5_H_12_]^−^;255.0293[M–H–Xyl–Rha–C_5_H_12_–CH_2_CO]^−^	√	√	√	demethylanhydroicaritin-3-O-rha(1-2)xyl
62	24.726	C_32_H_38_O_14_	645.22	1.70	499.1596[M–H–Rha]^−^;353.0993[M–H–2Rha]^−^;281.0448[M–H–2Rha–C_5_H_12_]^−^;255.0294[M–H–2Rha–C_5_H_12_–CH_2_CO]^−^	√	√	√	demethylanhydroicaritin-3-O-rha-(1-2)rha
63	24.845	C_36_H_42_O_17_	745.2363	1.88	583.1817[M–H–Glc]^−^;367.1188[M–H–Glc–Xyl(2OAc)]^−^;352.0941[M–H–Glc–Xyl(2OAc)–CH_3_·]^−^;323.0909[M–H–Glc–Xyl(2OAc)–CH_3_·–CHO]^−^	√	√	√	anhydroicaritin-3-O-xyl(2OAc)-7-O-glc
64	25.048	C_32_H_38_O_14_	645.2205	2.48	499.1587[M–H–Rha]^−^;353.0994[M–H–2Rha]^−^;323.0902[M–H–2Rha–CHO·]^−^;297.0967[M–H–2Rha–C_4_H_8_]^−^	√	√	√	demethylanhydroicaritin-3-O-rha-7-rha
65	25.133	C_27_H_30_O_11_	529.1720	0.94	383.1124[M–H–Rha]^−^;312.0632[M–H–Rha–C_4_H_8_–CH_3_·]^−^;297.0400[M–H–Rha–C_3_H_7_–CO–CH_3_·]^−^;296.0313[M–H–Rha–C_3_H_7_–2CO–CH_3_·]^−^	√	√	√	caohuoside C
66	25.152	C_29_H_32_O_11_	555.1856	−2.88	366.1094[M–H–Rha(OAc)–H·]·^−^;351.0856[M–H–Rha(OAc)– CH_3_·–H·]·^−^	-	-	√	anhydroicaritin-3-O-rha(OAc)
67	25.315	C_35_H_42_O_16_	717.2381	−2.65	555.1867[M–H–Glc]^−^;529.1711[M–H–Rha(OAc)]^−^;367.1174[M–H–Glc–Rha(OAc)]^−^	√	-	-	anhydroicaritin-3-O-rha(OAc)-7-O-glc
68	25.337	C_39_H_46_O_18_	801.2628	2.12	759.2488[M–H–OAc]^−^;555.1848[M–H–Glc(2OAc)]^−^;367.1185[M–H–Glc(OAc)–Rha(OAc)]^−^;352.0937[M–H–Glc(OAc)–Rha(OAc)–CH_3_·]^−^;311.0547[M–H–Glc(OAc)–Rha(OAc)–C_4_H_8_]^−^	-	√	-	anhydroicaritin-3-O-rha(OAc)-glc(2OAc)
69	25.380	C_45_H_56_O_23_	963.3155	1.56	801.2606[M–H–Glc]^−^;759.2482[M–H–Glc(OAc)]^−^;367.1173[M–H–Glc(2OAc)–Rha(OAc)–Glc]^−^;352.0937[M–H–Glc(2OAc)–Rha(OAc)–Glc–CH_3_·]^−^	-	√	-	caohuoside B
70	26.192	C_26_H_28_O_10_	499.1603	−1.40	353.1030[M–H–Rha]^−^;297.0657[M–H–Rha–C_4_H_8_]^−^	√	√	√	icarisoside A
71	26.508	C_45_H_56_O_23_	963.3176	3.74	801.1617[M–H–Glc]^−^;759.2498[M–H–Glc(OAc)]^−^;367.1174[M–H–Glc(2OAc)–Rha(OAc)–Glc]^−^;352.0937[M–H–Glc(2OAc)–Rha(OAc)–Glc–CH_3_·]^−^	-	√	-	caohuoside A
72	26.555	C_26_H_28_O_10_	499.1625	3.01	353.1027[M–H–Rha]^−^;297.0652[M–H–Rha–C_4_H_8_]^−^	√	√	√	demethylanhydroicaritin-3-O-rha
73	26.972	C_34_H_40_O_16_	703.2242	−0.28	541.1032[M–H–Glc]^−^;499.1396[M–H–Glc–OAc]^−^;353.0995[M–H–Glc–Rha(OAc)]^−^	-	√	-	demethylanhydroicaritin-3-O-rha(OAc)-glc
74	27.065	C_27_H_30_O_11_	529.1715	0	383.1104[M–H–Rha]^−^;327.0467[M–H–Rha–C_4_H_8_]^−^;283.0258[M–H–Rha–C_4_H_8_–CO_2_]^−^	√	√	√	caohuoside C isomer
75	27.132	C_26_H_28_O_11_	515.1547	−2.33	353.1021[M–H–Glc]^−^;297.0472[M–H–Glc–C_4_H_8_]^−^	-	√	√	demethylanhydroicaritin-7-O-glc
76	27.282	C_45_H_56_O_23_	963.3157	1.76	801.2633[M–H–Glc]^−^;759.2513[M–H–Glc(OAc)]^−^;529.1707[M–H–Glc(2OAc)–Rha(OAc)]^−^;367.1181[M–H–Glc(2OAc)–Rha(OAc)–Glc]^−^;352.0937[M–H–Glc(2OAc)–Rha(OAc)–Glc–CH_3_·]^−^;323.0921[M–H–Glc(2OAc)–Rha(OAc)–Glc–CH_3_·–CHO]^−^	-	√	-	korepimedoside B
77	27.528	C_33_H_40_O_15_	675.2314	2.96	513.1635[M–H–Glc]^−^;367.1178[M–H–Glc–Rha]^−^;352.0934[M–H–Glc–Rha–CH_3_·]^−^;323.0912[M–H–Glc–Rha–CH_3_·–CHO]^−^;311.0554[M–H–Glc–Rha–C_4_H_8_]^−^	√	√	√	icariin isomer
78	28.708	C_27_H_30_O_11_	529.1714	−0.19	367.1173[M–H–Glc]^−^;352.0933[M–H–Glc–CH_3_·]^−^;323.0905[M–H–Glc–CH_3_·–CHO]^−^	√	√	√	anhydroicaritin-3-O-glc
79	28.717	C_33_H_40_O_14_	659.2363	2.73	513.1637[M–H–Rha]^−^;366.1114[M–H–2Rha–H·]·^−^;351.0875[M–H–2Rha–CH_3_·–H·]·^−^;323.0919[M–H–2Rha–CH_3_·–CO–H·]·^−^;311.0556[M–H–2Rha–C_4_H_8_]^−^;295.0607[M–H–2Rha–CH_3_·–2CO–H·]·^−^;268.0367[M–H–2Rha–C_4_H_8_–C_2_H_2_O–H·]·^−^	√	√	√	2″-O-rhamnosyl icariside II
80	28.732	C_27_H_30_O_11_	529.1712	−0.57	367.1176[M–H–Glc]^−^;352.0859[M–H–Glc–CH_3_·]^−^;323.0916[M–H–Glc–CH_3_·–CHO]^−^;311.0556[M–H–Glc–C_4_H_8_]^−^	√	√	√	anhydroicaritin-7-O-glc
81	28.847	C_33_H_40_O_14_	659.237	3.79	513.1598[M–H–Rha]^−^;366.1101[M–H–2Rha –H·]·^−^;351.0661[M–H–2Rha–CH_3_·–H·]·^−^;323.0914[M–H–2Rha–CH_3_·–CO–H·]·^−^;311.0553[M–H–2Rha–C_4_H_8_]^−^;295.0605[M–H–2Rha–CH_3_·–2CO]^−^;268.0367[M–H–2Rha–C_4_H_8_–C_2_H_2_O –H·]·^−^	√	√	√	2″-O-rhamnosyl icariside II isomer
82	29.093	C_32_H_38_O_14_	645.2207	2.79	366.1116[M–H–Xyl–Rha –H·]·^−^;351.0877[M–H–Xyl–Rha–CH_3_·–H·]·^−^;323.0918[M–H–Xyl–Rha–CH_3_·–CO–H·]·^−^;311.0553[M–H–Xyl–Rha–C_4_H_8_]^−^;295.0611[M–H–Xyl–Rha–CH_3_·–2CO –H·]·^−^	√	√	√	sagittatoside B
83	29.232	C_36_H_42_O_17_	745.2359	1.34	703.2227[M–H–OAc]^−^;541.0365[M–H–Glc(OAc)]^−^;353.1022[M–H–Glc(OAc)–Rha(OAc)]^−^;325.1063[M–H–Glc(OAc)–Rha(OAc)–CO]^−^	-	√	-	demethylanhydroicaritin-3-O-rha(OAc)-glc(OAc)
84	29.705	C_33_H_40_O_14_	659.234	−0.76	513.1694[M–H–Rha]^−^;366.1096[M–H–2Rha –H·]·^−^;351.0859[M–H–2Rha–CH_3_·–H·]·^−^;323.0908[M–H–2Rha–CH_3_·–CO–H·]·^−^;311.0550[M–H–2Rha–C_4_H_8_]^−^;295.0606[M–H–2Rha–CH_3_·–2CO –H·]·^−^	√	√	√	anhydroicaritin-3-O-rha-7-O-rha
85	30.048	C_35_H_42_O_16_	717.2426	3.63	675.2275[M–H–OAc]^−^;513.1716[M–H–Glc(OAc)]^−^;367.1175[M–H–Glc(OAc)–Rha]^−^;352.0931[M–H–Glc(OAc)–Rha–CH_3_·]^−^;323.0898[M–H–Glc(OAc)–Rha–CH_3_·–CHO]^−^;279.1010[M–H–Glc(OAc)–Rha–CH_3_·–CHO–CO_2_]^−^	√	-	-	anhydroicaritin-3-O-rha-glc(OAc)
^a^ 86	31.183	C_27_H_30_O_10_	513.1783	3.31	366.1109[M–H–Rha–H·]·^−^;351.0879[M–H–Rha–CH_3_·–H·]·^−^;337.1072[M–H–Rha–CHO–H·]·^−^;323.0927[M–H–Rha–CH_3_·–CO–H·]·^−^;311.0558[M–H–Rha–C_4_H_8_]^−^;295.0609[M–H–Rha–CH_3_·–CO –H·]·^−^;279.1022[M–H–Rha–CH_3_·–CO_2_ –H·]·^−^	√	√	√	baohuoside I or icariin II
87	31.378	C_34_H_42_O_15_	689.2446	−0.72	513.1742[M–H–Glc–CH_2_]^−^;367.1139[M–H–Glc–CH_3_·–Rha]^−^	√	√	√	anhydroicaritin-3-O-rha-glc-CH_3_
* 88	31.937	C_33_H_38_O_14_	657.2201	1.83	513.1760[M–H–C_6_H_8_O_4_]^−^;367.1179[M–H–C_6_H_8_O_4_–Rha]^−^;352.0932[M–H–C_6_H_8_O_4_–Rha–CH_3_·]^−^;323.0910[M–H–C_6_H_8_O_4_–Rha–CH_3_·–CHO]^−^	√	√	√	anhydroicaritin-3-O-rha-C_6_H_8_O_4_
89	32.025	C_37_H_44_O_17_	759.2529	3.03	555.2210[M–H–Glc(OAc)]^−^;367.1194[M–H–Glc(OAc)–Rha(OAc)]^−^;352.0946[M–H–Glc(OAc)–Rha(OAc)–CH_3_·]^−^;323.0914[M–H–Glc(OAc)–Rha(OAc)–CH_3_·–CHO]^−^;311.0554[M–H–Glc(OAc)–Rha(OAc)–C_4_H_8_]^−^	-	√	-	anhydroicaritin-3-O-rha(1-4OAc)-glc(1-4OAc)
90	32.535	C_34_H_42_O_14_	673.2502	0	513.1752[M–H–Rha–CH_2_]^−^;367.1139[M–H–Rha–CH_2_–Rha]^−^	√	√	√	anhydroicaritin-3-O-rha-CH_3_-rha
91	33.305	C_20_H_18_O_6_	353.1026	−1.42	337.0697[M–H–H_2_O]^−^;281.0246[M–H–H_2_O–C_4_H_8_]^−^;153.0164[^1,3^A–isopentenyl]^−^	√	-	√	aglycone
92	33.432	C_39_H_46_O_18_	801.2636	3.12	759.2500[M–H–OAc]^−^;555.2135[M–H–Glc(2OAc)]^−^;367.1186[M–H–Glc(2OAc)–Rha(OAc)]^−^;352.0938[M–H–Glc(2OAc)–Rha(OAc)–CH_3_·]^−^;323.0909[M–H–Glc(2OAc)–Rha(OAc)–CH_3_·–CHO]^−^;311.0554[M–H–Glc(2OAc)–Rha(OAc)–C_4_H_8_]^−^;295.0608[M–H–Glc(2OAc)–Rha(OAc)–CH_3_·–2CO]^−^;279.1023[M–H–Glc(2OAc)–Rha(OAc)–CH_3_·–2CO–H_2_O]^−^	-	√	-	anhydroicaritin-3-O-rha(OAc)-glc(2OAc)
93	33.545	C_35_H_42_O_16_	717.2416	2.23	513.1721[M–H–Glc(OAc)]^−^;366.1093[M–H–Glc(OAc)–Rha –H·]·^−^;351.0860[M–H–Glc(OAc)–Rha–CH_3_·–H·]·^−^;323.0916[M–H–Glc(OAc)–Rha–CH_3_·–CO –H·]·^−^;311.0552[M–H–Glc(OAc)–Rha–C_4_H_8_]^−^;295.0610[M–H–Glc(OAc)–Rha·–CH_3_·–2CO–H·]·^−^	-	√	-	anhydroicaritin-3-O-rha-glc(OAc)
94	33.655	C_29_H_32_O_11_	555.187	−0.36	366.1093[M–H–Glc(OAc)–H·]·^−^;351.0853[M–H–Glc(OAc)–CH_3_·–H·]·^−^;323.0912[M–H–Glc(OAc)–CH_3_·–CO –H·]·^−^;311.0562[M–H–Glc(OAc)–C_4_H_8_]^−^	√	-	√	anhydroicaritin-3-O-rha(OAc)
95	33.853	C_39_H_46_O_18_	801.2624	1.62	759.2450[M–H–OAc]^−^;555.2543[M–H–Glc(2OAc)]^−^;367.1176[M–H–Glc(2OAc)–Rha(OAc)]^−^;352.0938[M–H–Glc(2OAc)–Rha(OAc)–CH_3_·]^−^;323.0911352.0938[M–H–Glc(2OAc)–Rha(OAc)–CH_3_·–CHO]^−^;311.0554[M–H–Glc(2OAc)–Rha(OAc)–C_4_H_8_]^−^;295.0612[M–H–Glc(2OAc)–Rha(OAc)–CH_3_·–CHO–CO]^−^;279.1019[M–H–Glc(2OAc)–Rha(OAc)–CH_3_·–CHO–CO–H_2_O]^−^	-	√	-	anhydroicaritin-3-O-rha(OAc)-glc(2OAc)
96	35.245	C_25_H_26_O_6_	421.1663	1.42	365.1022[M–H–C_4_H_8_]^−^;309.0555[M–H–C_4_H_8_–C_4_H_8_]^−^;151.0031[^1,3^A–isopenteny]^−^	√	√	√	epimedokoreanin B
97	35.568	C_21_H_20_O_6_	367.1194	1.91	352.2177[M–H–CH_3_·]^−^;219.0614[^1,3^A]^−^	√	√	√	anhydroicaritin
98	35.975	C_25_H_26_O_5_	405.1714	1.72	349.1060[M–H–C_4_H_8_]^−^;293.0601[M–H–C_4_H_8_–C_4_H_8_]^−^	-	-	√	4′,5,7-trihydroxy-5′,8-diisopentenylflavone or isomer
99	36.178	C_25_H_26_O_5_	405.171	0.74	349.1063[M–H–C_4_H_8_]^−^;293.0601[M–H–C_4_H_8_–C_4_H_8_]^−^	-	-	√	4′,5,7-trihydroxy-5′,8-diisopentenyl-flavone or isomer

Note: ^a^: consolidated with standard reference; *: neutral loss of 144 Da; Esa: *Epimedium sagittatum* (Sieb. *et* Zucc.) Maxim; Eko: *Epimedium koreanum* Nakai; Epu: *Epimedium pubescens* Maxim.

## Data Availability

No new data were created or analyzed in this study. Data sharing is not applicable to this article.

## Supplementary Materials

The following materials are available online: the in–house data library for the chemical compounds identified from *Epimedium* genus as supporting information.
