# Supplementary material for: Direct Flavonoid-Focused Chemical Comparison among Three Epimedium Plants by Online Liquid Extraction–High Performance Liquid Chromatography–Tandem Mass Spectrometry
Source: Molecules. 2021 Mar 10;26(6):1520. doi: 10.3390/molecules26061520 (PMC7998785; doi:10.3390/molecules26061520)
Supplement: Supplementary file 1 [file molecules-26-01520-s001.pdf]

Supporting Information

Direct Flavonoid-Focused Chemical Comparison Among Three *Epimedium* Plants by Online Liquid Extraction–High Performance Liquid Chromatography–Tandem Mass Spectrometry

Xia Xu [1], Ting Li [1], Ke Zhang [1], Yan Cao [1], Li Liu [2], Shilin Zhang [2], Pengfei Tu [1], Yuelin Song [1], Yunfang Zhao [1,*] and Jun Li [1,*]

1 Modern Research Center for Traditional Chinese Medicine, School of Chinese Materia Medica, Beijing University of Chinese Medicine, Beijing 100029, China; Xkilly1119@163.com (X.X.); 18201353145@163.com (T.L.); zk_5353@163.com (K.Z.); cyan199409@163.com (Y.C.); pengfeitu@163.com (P.T.); syltwc2005@163.com (Y.S.)

2 Guizhou Hanfang Pharmaceutical Co. Ltd., Guiyang 550014, China; dcxliuli@163.com (L.L.); zhang_shi_lin2021@163.com (S.Z.)

* Correspondence: yunfang.zhao@163.com (Y.Z.); drlj666@163.com (J.L.); Tel./Fax.: +86-010-6428-6350 (Y.Z.)

Table S1. The in-house data library for the chemical components identified from *Epimedium* genus.

No.	Molecular formula	Exact Mass	Integer/Da	Mass defect/ mDa	Identity	Reference
1	$C_{13}H_8O_6$	260.0321	260	32.10	1,3,5,8-tetrahydroxy xanthone	1
2	$C_{15}H_{12}O_4$	256.0736	256	73.60	liquiritigenin	2
3	$C_{15}H_{12}O_4$	256.0736	256	73.60	isoliquirigenin	3
4	$C_{15}H_{10}O_5$	270.0528	270	52.80	apigenin	4
5	$C_{15}H_{10}O_6$	286.0477	286	47.70	kaempferol	5
6	$C_{15}H_{10}O_6$	286.0477	286	47.70	luteolin	5
7	$C_{16}H_{12}O_6$	300.0634	300	63.40	6-demethoxy-7-methylcapillarisin	6
8	$C_{16}H_{12}O_6$	300.0634	300	63.40	chrysoeriol	7
9	$C_{15}H_{10}O_7$	302.0427	302	42.70	quercetin	5
10	$C_{16}H_{14}O_6$	302.0790	302	79.00	1-hydroxy-3,5,8-trimethoxy xanthone	1
11	$C_{16}H_{12}O_7$	316.0583	316	58.30	tricetin-3′-methyl ether	2
12	$C_{17}H_{14}O_7$	330.0740	330	74.00	tricin	8
13	$C_{18}H_{14}O_7$	330.0740	330	74.00	5-hydroxy-6,7-methylene-dioxyflavone	9
14	$C_{20}H_{16}O_5$	336.0998	336	99.80	yinyanghuo C	10
15	$C_{20}H_{16}O_5$	336.0998	336	99.80	yinyanghuo E	10
16	$C_{20}H_{18}O_5$	338.1154	338	115.40	yinyanghuo D	10
17	$C_{20}H_{16}O_6$	352.0947	352	94.70	epimedonin B	10
18	$C_{21}H_{20}O_5$	352.1311	352	131.10	8-(3,3-dimethylallyl)-5,7-dihydroxy-4′-methoxyflavonol 3-[O-3,5-di-deoxy-2-C-(ethoxycarbonyl)-β-D-erythropento-furanosyl-(1→2)-α-L-rha] 7-(β-D-glc)	2
19	$C_{20}H_{18}O_6$	354.1103	354	110.30	demethylanhydroicaritin	4
20	$C_{20}H_{18}O_6$	354.1103	354	110.30	2-(p-hydroxyphenoxy)-5, 7-dihydroxy-6-iso-pentenyl chromone	11
21	$C_{20}H_{18}O_6$	354.1103	354	110.30	epimedokoreanin D	12
22	$C_{20}H_{18}O_6$	354.1103	354	110.30	8-prenylkaempferol	13
23	$C_{20}H_{18}O_6$	354.1103	354	110.30	6-demthoxy-7-isopentenylcapillarisin	6
24	$C_{20}H_{18}O_6$	354.1103	354	110.30	aglycone A	14
25	$C_{21}H_{20}O_6$	368.1260	368	126.00	epimedokoreanin D	2
26	$C_{21}H_{20}O_6$	368.1260	368	126.00	anhydroicaritin	2
27	$C_{21}H_{20}O_6$	368.1260	368	126.00	epimedonin A	15
28	$C_{21}H_{22}O_6$	370.1416	370	141.60	6-demethoxy-4′-methyl-8-isopentenylcapil-larisin	6
29	$C_{21}H_{20}O_7$	384.1209	384	120.90	aglycone C	14
30	$C_{21}H_{20}O_7$	384.1209	384	120.90	2″-hydroxy-β-anhydroicaritin	2
31	$C_{21}H_{22}O_7$	384.1209	384	120.90	wushanicaritin	16

32	$C_{21}H_{22}O_7$	384.1209	384	120.90	5,7-dihydroxy-2-(p-hydroxyphenoxy)-6-prenyichromone	17
33	$C_{22}H_{22}O_7$	398.1366	398	136.60	baohuosu	18
34	$C_{22}H_{24}O_7$	400.1522	400	152.20	brevicomin	19
35	$C_{22}H_{30}O_7$	406.1992	406	199.20	3″-ethyoxylicartin	20
36	$C_{25}H_{26}O_5$	406.1780	406	178.00	5,7,4-trihydroxy-8,3-diprenylflavone	21
37	$C_{25}H_{26}O_5$	406.1780	406	178.00	4′5,7-trihydroxy-5′,8-di isopentenyl-flavone	3
38	$C_{25}H_{22}O_6$	418.1416	418	141.60	epimedonin E	22
39	$C_{25}H_{24}O_6$	420.1573	420	157.30	yinyanghuo A	10
40	$C_{25}H_{24}O_6$	420.1573	420	157.30	epimedonin C	22
41	$C_{25}H_{24}O_6$	420.1573	420	157.30	epimedonin F	22
42	$C_{25}H_{26}O_6$	422.1729	422	172.90	yinyanghuo B	10
43	$C_{25}H_{26}O_6$	422.1729	422	172.90	epimedokoreanin B	3
44	$C_{25}H_{26}O_6$	422.1729	422	172.90	broussoflavonol F	3
45	$C_{21}H_{20}O_{10}$	432.1056	432	105.60	kaempferol-3-O-α-rha	18
46	$C_{26}H_{28}O_6$	436.1886	436	188.60	epimedonin D	15
47	$C_{25}H_{26}O_7$	438.1679	438	167.90	breviflavone A	23
48	$C_{25}H_{26}O_7$	438.1679	438	167.90	breviflavone B	23
49	$C_{25}H_{26}O_7$	438.1679	438	167.90	desmethylicaritin	20
50	$C_{21}H_{20}O_{11}$	448.1006	448	100.60	quercetin -3-O- rha	24
51	$C_{21}H_{20}O_{11}$	448.1006	448	100.60	astragalin	25
52	$C_{21}H_{20}O_{11}$	448.1006	448	100.60	trifolin	18
53	$C_{21}H_{20}O_{11}$	448.1006	448	100.60	kaempferol-3-O-gal	3
54	$C_{21}H_{20}O_{11}$	448.1006	448	100.60	kaempferol-7-O-glc	3
55	$C_{21}H_{20}O_{11}$	448.1006	448	100.60	quercetin-3-O-rha	3
56	$C_{25}H_{24}O_8$	452.1471	452	147.10	epimedokoreanin A	2
57	$C_{25}H_{26}O_8$	454.1628	454	162.80	epimedokoreanin C	26
58	$C_{21}H_{20}O_{12}$	464.0955	464	95.50	hyperoside	24
59	$C_{21}H_{22}O_{12}$	464.0955	464	95.50	dihydroquercetin-7-O-glc	24
60	$C_{21}H_{20}O_{12}$	464.0955	464	95.50	hyperoside	3
61	$C_{21}H_{20}O_{12}$	464.0955	464	95.50	quercetin-3-O-gal	3
62	$C_{21}H_{20}O_{12}$	464.0955	464	95.50	quercetin-7-O-glc	3
63	$C_{21}H_{20}O_{13}$	480.0904	480	90.40	isoarbayonin	18
64	$C_{21}H_{20}O_{13}$	480.0904	480	90.40	myricetin-3-O-glc	3
65	$C_{26}H_{26}O_{10}$	498.1526	498	152.60	icarisoside E	27
66	$C_{26}H_{28}O_{10}$	500.1682	500	168.20	baohuoside II	25
67	$C_{26}H_{28}O_{10}$	500.1682	500	168.20	ikarisoside A	18
68	$C_{26}H_{28}O_{10}$	500.1682	500	168.20	demethylanhydroicaritin-3-O-rha	3
69	$C_{27}H_{28}O_{10}$	512.1682	512	168.20	acuminatoside	2
70	$C_{27}H_{30}O_{10}$	514.1839	514	183.90	baohuoside I	3
71	$C_{27}H_{30}O_{10}$	514.1839	514	183.90	icariside II	3
72	$C_{27}H_{30}O_{10}$	514.1839	514	183.90	anhydroicaritin-3-O-rha	3
73	$C_{26}H_{28}O_{11}$	516.1632	516	163.20	epimedoside C	18
74	$C_{26}H_{28}O_{11}$	516.1632	516	163.20	demethylanhydroicaritin-7-O-glc	3
75	$C_{26}H_{30}O_{11}$	518.1788	518	178.80	dihydrodemethylicaritin-7-O-glc	3
76	$C_{27}H_{28}O_{11}$	528.1632	528	163.20		28
77	$C_{27}H_{30}O_{11}$	530.1788	530	178.80	caohuoside C	18
78	$C_{27}H_{30}O_{11}$	530.1788	530	178.80	icariside I	24
79	$C_{27}H_{30}O_{11}$	530.1788	530	178.80	wushan icariin	2
80	$C_{27}H_{30}O_{11}$	530.1788	530	178.80	caohuoside D	29
81	$C_{27}H_{30}O_{11}$	530.1788	530	178.80	koreanoside E	30
82	$C_{27}H_{30}O_{11}$	530.1788	530	178.80	anhydroicaritin-7-O-glc or isomer	3
83	$C_{27}H_{30}O_{11}$	530.1788	530	178.80	3′-hydroxyicariine-7-O-rha or isomer	3
84	$C_{27}H_{30}O_{11}$	530.1788	530	178.80	icariin I	25
85	$C_{26}H_{28}O_{12}$	532.1581	532	158.10		31
86	$C_{27}H_{32}O_{11}$	532.1945	532	194.50	icaritin-3-O-rha or isomer	24
87	$C_{28}H_{30}O_{11}$	542.1788	542	178.80	ikarisoside D	27
88	$C_{27}H_{30}O_{12}$	546.1737	546	173.70	3′-hydroxyicariine-3-O-glc	3
89	$C_{27}H_{32}O_{12}$	548.1894	548	189.40	maohuoside A	2
90	$C_{27}H_{34}O_{12}$	550.2050	550	205.00	acumination	32
91	$C_{31}H_{20}O_{10}$	552.1056	552	105.60	bilobetin	17
92	$C_{29}H_{32}O_{11}$	556.1945	556	194.50	anhydroicaritin-3-O-rha(OAc)	3

93	$C_{29}H_{32}O_{11}$	556.1945	556	194.50	anhydroicaritin-3-O-rha(OAc) isomer	3
94	$C_{28}H_{34}O_{12}$	562.2050	562	205.00	caohuoside D	2
95	$C_{28}H_{34}O_{12}$	562.2050	562	205.00	caohuoside F	2
96	$C_{26}H_{28}O_{14}$	564.1479	564	147.90	kaempferol-7-O-rha-xyl	3
97	$C_{32}H_{22}O_{10}$	566.1213	566	121.30	ginkgetin	17
98	$C_{32}H_{22}O_{10}$	566.1213	566	121.30	isoginkgetin	17
99	$C_{27}H_{30}O_{14}$	578.1636	578	163.60	kaempferol -3-O-di-rha	24
100	$C_{27}H_{30}O_{14}$	578.1636	578	163.60	kaempferitrin	18
101	$C_{27}H_{30}O_{14}$	578.1636	578	163.60	sempervirenoside B	33
102	$C_{27}H_{30}O_{14}$	578.1636	578	163.60	kaempferol-3-O-rha-7-O-rha	3
103	$C_{27}H_{30}O_{14}$	578.1636	578	163.60	kaempferol-3-O-rha-rha	3
104	$C_{26}H_{28}O_{15}$	580.1428	580	142.80	quercetin-3-O-rha-xyl	3
105	$C_{28}H_{36}O_{13}$	580.2156	580	215.60	icariside E7	4
106	$C_{31}H_{36}O_{11}$	584.2258	584	225.80	3′,4′,5,7-tetrahydroxy-5′,8-diisopentenyl-flavone-7-O-glc or broussoflavonol F	3
107	$C_{27}H_{30}O_{15}$	594.1585	594	158.50	quercetin-3,7-O-di-rha	24
108	$C_{27}H_{30}O_{15}$	594.1585	594	158.50	quercetin-3-O-glc-7-O-rha	3
109	$C_{29}H_{32}O_{15}$	620.1741	620	174.10	sutchuenoside A	34
110	$C_{29}H_{32}O_{15}$	620.1741	620	174.10	kaempferol-3-O-rha-7-O-rha(OAc)	3
111	$C_{29}H_{34}O_{15}$	622.1898	622	189.80	wushanicariin	35
112	$C_{31}H_{36}O_{14}$	632.2105	632	210.50	icarisoside F	25
113	$C_{31}H_{36}O_{14}$	632.2105	632	210.50	demethylanhydroicaritin-3-O-rhamnopyranosyl-xylopyranoside	24
114	$C_{33}H_{36}O_{13}$	640.2156	640	215.60		28
115	$C_{32}H_{34}O_{14}$	642.1949	642	194.90	demethylanhydroicaritin-3-O-rha-dideoxy-furanose	3
116	$C_{32}H_{38}O_{14}$	646.2262	646	226.20	2″-O-rhamnosylikarisoside A	2
117	$C_{32}H_{38}O_{14}$	646.2262	646	226.20	baohuoside III	36
118	$C_{32}H_{38}O_{14}$	646.2262	646	226.20	baohuoside IV	36
119	$C_{32}H_{38}O_{14}$	646.2262	646	226.20	sagittatoside B	3
120	$C_{32}H_{38}O_{14}$	646.2262	646	226.20	anhydroicaritin-3-O-rha-(1-2)-xyl	3
121	$C_{32}H_{38}O_{14}$	646.2262	646	226.20	demethylanhydroicaritin-3-O-rha-(1-2)rha	3
122	$C_{31}H_{36}O_{15}$	648.2054	648	205.40	demethylanhydroicaritin-3-O-glc(1-2)xyl	3
123	$C_{33}H_{36}O_{14}$	656.2105	656	210.50	sutchuenmedin B	34
124	$C_{33}H_{38}O_{14}$	658.2262	658	226.20	anhydroicaritin-3-O-rhamnopyranosyl-furan acid or isomer	24
125	$C_{33}H_{38}O_{14}$	658.2262	658	226.20	sutchuenmedin A	37
126	$C_{33}H_{38}O_{14}$	658.2262	658	226.20		28
127	$C_{33}H_{38}O_{14}$	658.2262	658	226.20		31
128	$C_{33}H_{40}O_{14}$	660.2418	660	241.80	2″-O-rhamnosyl icariside II	24
129	$C_{33}H_{40}O_{14}$	660.2418	660	241.80	2″-O-rhamnosyl icarisideII isomer	24
130	$C_{33}H_{40}O_{14}$	660.2418	660	241.80	anhydroicaritin-3-O-rha-(1-2)-rha	3
131	$C_{33}H_{40}O_{14}$	660.2418	660	241.80	anhydroicaritin-3-O-rha-7-O-rha	3
132	$C_{32}H_{38}O_{15}$	662.2211	662	221.10	epimedoside A	24
133	$C_{32}H_{38}O_{15}$	662.2211	662	221.10	icarisoside B or isomer	25
134	$C_{32}H_{38}O_{15}$	662.2211	662	221.10	ikarisoside B	25
135	$C_{32}H_{38}O_{15}$	662.2211	662	221.10	epimedoside A	18
136	$C_{32}H_{38}O_{15}$	662.2211	662	221.10	demethylanhydroicaritin-3-O-rha-(1-2)glc or isomer	3
137	$C_{32}H_{38}O_{15}$	662.2211	662	221.10	demethylanhydroicaritin-3-O-rha-7-O-glc or isomer	3
138	$C_{32}H_{38}O_{15}$	662.2211	662	221.10	3′-hydroxyicariine-3-O-rha-xyl	3
139	$C_{32}H_{38}O_{15}$	662.2211	662	221.10	3′-hydroxyicariine-7-O-rha-xyl or isomer	3
140	$C_{33}H_{38}O_{15}$	674.2211	674	221.10	4, 5, 7-trihydroxyl-8-(3, 3-dimethylallyl)-flavonol 3-O-xyl (1→3)-4-Oacetyl-rha	2
141	$C_{33}H_{38}O_{15}$	674.2211	674	221.10	anhydroicaritin-3-o-rha-(1-4)-didedoxy-furanose-7-O-glc	3
142	$C_{33}H_{38}O_{15}$	674.2211	674	221.10	anhydroicaritin-3-O-rha-(1-2)-didedoxy-furanose-7-O-glc	3
143	$C_{33}H_{40}O_{15}$	676.2367	676	236.70	icariin	2
144	$C_{33}H_{40}O_{15}$	676.2367	676	236.70	3′-hydroxyicariine-3-di-O-rha	24
145	$C_{33}H_{40}O_{15}$	676.2367	676	236.70	baohuoside VII	24

146	$C_{33}H_{40}O_{15}$	676.2367	676	236.70	sagittatoside A	24
147	$C_{33}H_{40}O_{15}$	676.2367	676	236.70	baohuoside VII	24
148	$C_{33}H_{40}O_{15}$	676.2367	676	236.70	4-methoxyl-3, 5, 7-trihydroxyl-8-(3, 3- di-methylallyl)-flavonol 3-O-rha (1→2)-rha	2
149	$C_{33}H_{40}O_{15}$	676.2367	676	236.70	sagittasine C	38
150	$C_{33}H_{40}O_{15}$	676.2367	676	236.70	anhydroicaritin-3-O-rha-glc	3
151	$C_{33}H_{40}O_{15}$	676.2367	676	236.70	3′-hydroxyicariine-3-O-rha-rha	3
152	$C_{33}H_{40}O_{15}$	676.2367	676	236.70	3″-hydroxyicariine-3-O-rha-7-O-rha or iso-mer	3
153	$C_{32}H_{38}O_{16}$	678.2160	678	216.00	dihydroanhydroicaritin-3-O-rha-7-O-glc	24
154	$C_{32}H_{38}O_{16}$	678.2160	678	216.00	hexandraside E or isomer	24
155	$C_{32}H_{38}O_{16}$	678.2160	678	216.00	hexandraside E	18
156	$C_{32}H_{38}O_{16}$	678.2160	678	216.00	4, 5-dihydroxyl-8-(3, 3-dimethylallyl)-flavo-nol 3-O-[xyl (1→3)-rha]-7-O-glc	2
157	$C_{32}H_{38}O_{16}$	678.2160	678	216.00	8-(3, 3-dimethylallyl)-4′, 5, 7-trihydroxyfla-vonol 7-[O-β-D-glc-(1→2)-β-D-glc]	2
158	$C_{32}H_{38}O_{16}$	678.2160	678	216.00	demethylanhydroicaritin-3-O-glc-7-O-glc	3
159	$C_{32}H_{38}O_{16}$	678.2160	678	216.00	dihydrodemethylanhydroicaritin-7-O-sophoroside	3
160	$C_{33}H_{42}O_{15}$	678.2524	678	252.40	wanepimedoside A	2
161	$C_{32}H_{40}O_{16}$	680.2316	680	231.60	dihydrodemethylicaritin-7-O-glc-glc	3
162	$C_{34}H_{40}O_{15}$	688.2367	688	236.70		28
163	$C_{33}H_{38}O_{16}$	690.2160	690	216.00		31
164	$C_{33}H_{40}O_{16}$	692.2316	692	231.60	sagittasine C	24
165	$C_{33}H_{40}O_{16}$	692.2316	692	231.60	anhydroicaritin-3,7-di-O-glc	18
166	$C_{33}H_{40}O_{16}$	692.2316	692	231.60	kaempferin-3-O-α-rha	4
167	$C_{33}H_{40}O_{16}$	692.2316	692	231.60	cuhuoside	2
168	$C_{33}H_{40}O_{16}$	692.2316	692	231.60	3′-hydroxyicariine-3-O-rha-glc	3
169	$C_{32}H_{38}O_{17}$	694.2109	694	210.90		31
170	$C_{32}H_{38}O_{17}$	694.2109	694	210.90		31
171	$C_{35}H_{42}O_{15}$	702.2524	702	252.40	acetylicariin	2
172	$C_{34}H_{40}O_{16}$	704.2316	704	231.60	demethylanhydroicaritin-3-O-rha(OAc)-glc	3
173	$C_{32}H_{38}O_{18}$	710.2058	710	205.80	kaempferol-3-O-rha-xyl-7-O-glc	3
174	$C_{35}H_{42}O_{16}$	718.2473	718	247.30	sagittatoside C	3
175	$C_{35}H_{42}O_{16}$	718.2473	718	247.30	anhydroicaritin-3-O-glc(OAc)-7-O-rha	3
176	$C_{35}H_{42}O_{16}$	718.2473	718	247.30	anhydroicaritin-3-O-rha(OAc)-7-O-glc	3
177	$C_{34}H_{40}O_{17}$	720.2266	720	226.60		31
178	$C_{35}H_{42}O_{17}$	734.2422	734	242.20		28
179	$C_{35}H_{42}O_{17}$	734.2422	734	242.20		31
180	$C_{34}H_{42}O_{18}$	738.2371	738	237.10		14
181	$C_{33}H_{40}O_{19}$	740.2164	740	216.40	kaempferol-3-O-rha-glc-7-O-rha	24
182	$C_{33}H_{40}O_{19}$	740.2164	740	216.40	kaempferol-3-O-rha-glc-7-O-glc	3
183	$C_{36}H_{42}O_{17}$	746.2422	746	242.20	anhydroicaritin-3-O-xyl(2OAc)-7-O-glc	3
184	$C_{36}H_{42}O_{17}$	746.2422	746	242.20	demethylanhydroicaritin-3-O-rha(OAc)-glc(OAc)	3
185	$C_{37}H_{44}O_{17}$	760.2579	760	257.90	epimedoside	2
186	$C_{37}H_{44}O_{17}$	760.2579	760	257.90	anhydroicaritin-3-o-rha(1-4OAc)-glc(1-4OAc)	3
187	$C_{37}H_{44}O_{17}$	760.2579	760	257.90	korepimedoside A isomer	39
188	$C_{36}H_{42}O_{18}$	762.2371	762	237.10		28
189	$C_{38}H_{44}O_{18}$	788.2528	788	252.80		31
190	$C_{37}H_{46}O_{19}$	794.2633	794	263.30	epimedoside D	40
191	$C_{37}H_{46}O_{19}$	794.2633	794	263.30	epimedoside E	24
192	$C_{37}H_{46}O_{19}$	794.2633	794	263.30		31
193	$C_{39}H_{46}O_{18}$	802.2684	802	268.40	korepimeoside A	39
194	$C_{39}H_{46}O_{18}$	802.2684	802	268.40	korepimeoside B	39
195	$C_{39}H_{46}O_{18}$	802.2684	802	268.40	anhydroicaritin-3-O-rha(OAc)-glc(2OAc)	3
196	$C_{39}H_{46}O_{18}$	802.2684	802	268.40	anhydroicaritin-3-O-rha(OAc)-glc(2OAc) isomer	3
197	$C_{38}H_{46}O_{19}$	806.2633	806	263.30		28
198	$C_{38}H_{48}O_{19}$	808.2790	808	279.00	diphylloside B	24
199	$C_{38}H_{48}O_{19}$	808.2790	808	279.00	diphylloside A	18

200	$C_{38}H_{48}O_{19}$	808.2790	808	279.00	epimedin B	4
201	$C_{38}H_{48}O_{19}$	808.2790	808	279.00	baohuoside V	36
202	$C_{38}H_{48}O_{19}$	808.2790	808	279.00	anhydroicaritin-3-O-rha-(1-4)-xyl-7-O-glc	3
203	$C_{38}H_{48}O_{19}$	808.2790	808	279.00	demethylanhydroicaritin-3-O-rha-rha-7-O-glc	3
204	$C_{38}H_{48}O_{19}$	808.2790	808	279.00	epimedin B isomer	28
205	$C_{37}H_{46}O_{20}$	810.2582	810	258.20		31
206	$C_{37}H_{46}O_{20}$	810.2582	810	258.20		31
207	$C_{39}H_{49}NO_{18}$	819.2950	819	295.00	3-acetyl-3,5,12-trihydroxy-10-methoxy-6,11-dioxo- 1,2,3,4,6,11-hexahydro-1-tetracenyl,3,6-trideoxy-3-{[4-ethoxy-4-(β-D-glccopyranuronosyloxy)butyl]amino}- L-xylo-hexopyranoside	18
208	$C_{39}H_{49}NO_{18}$	819.2950	819	295.00		14
209	$C_{39}H_{48}O_{19}$	820.2790	820	279.00	anhydroicaritin-3-O-rha (1-2)-furan acid-7-O-glc	18
210	$C_{39}H_{48}O_{19}$	820.2790	820	279.00	anhydroicaritin-3-O-rha-(1-3)-furan acid-7-O-glc	4
211	$C_{39}H_{48}O_{19}$	820.2790	820	279.00	anhydroicaritin-3-O-rha (1-4) -furan acid-7-O-glc	24
212	$C_{39}H_{48}O_{19}$	820.2790	820	279.00	anhydroicaritin-3-O-glc (1-2) -furan acid-7-O-rha	24
213	$C_{39}H_{48}O_{20}$	820.2790	820	279.00	4, 5-dihydroxyl-8-(3,3-dimethylallyl)-flavonol-3-O-[xyl (1→3)-4-O-acetylrha]-7-O-glc	2
214	$C_{39}H_{48}O_{20}$	820.2790	820	279.00	demethylanhydroicaritin-3-O-rha-xyl-7-O-glc(OAc)	3
215	$C_{39}H_{50}O_{19}$	822.2946	822	294.60	baohuoside VI	25
216	$C_{38}H_{46}O_{20}$	822.2582	822	258.20		14
217	$C_{39}H_{50}O_{19}$	822.2946	822	294.60	baohuoside VI	18
218	$C_{39}H_{50}O_{19}$	822.2946	822	294.60	epimedin C	4
219	$C_{39}H_{50}O_{19}$	822.2946	822	294.60	hexandraside D	24
220	$C_{39}H_{50}O_{19}$	822.2946	822	294.60	anhydroicaritin-3-O-rha-(1-4)-rha-7-O-glc	3
221	$C_{39}H_{50}O_{19}$	822.2946	822	294.60	anhydroicaritin-3-O-rha-glc-7-O-rha	3
222	$C_{39}H_{50}O_{19}$	822.2946	822	294.60	epimedin C isomer	14
223	C_{38} $H_{48}O_{20}$	824.2739	824	273.90	rouhuoside	24
224	$C_{38}H_{48}O_{20}$	824.2739	824	273.90	diphylloside B	18
225	$C_{38}H_{48}O_{20}$	824.2739	824	273.90	ikarisoside C	12
226	$C_{40}H_{46}NO_{18}$	828.2715	828	271.50		14
227	C_{39} $H_{48}O_{20}$	836.2739	836	273.90	demethylanhydroicaritin-7-O-glc-3-O-acetylated rha-xyl	24
228	$C_{39}H_{48}O_{21}$	836.2739	836	273.90		14
229	$C_{39}H_{48}O_{21}$	836.2739	836	273.90		31
230	$C_{39}H_{48}O_{21}$	836.2739	836	273.90		31
231	$C_{39}H_{48}O_{21}$	836.2739	836	273.90	anhydroicaritin-3-O-rha-glccuronic acid-7-O-glc	24
232	$C_{39}H_{50}O_{20}$	838.2895	838	289.50	hexandraside F	3
233	C_{39} $H_{50}O_{20}$	838.2895	838	289.50	3′-hydroxyicariine-3-di-O-rha-7-O-glc	24
234	C_{39} $H_{50}O_{20}$	838.2895	838	289.50	hexandraside F isomer	24
235	$C_{39}H_{50}O_{20}$	838.2895	838	289.50	sagittasine B	3
236	$C_{39}H_{50}O_{20}$	838.2895	838	289.50	epimedin A	25
237	$C_{39}H_{50}O_{20}$	838.2895	838	289.50	hexandraside F	18
238	$C_{39}H_{50}O_{20}$	838.2895	838	289.50	epimedin A isomer	2
239	$C_{39}H_{50}O_{20}$	838.2895	838	289.50	morphoside B	2
240	$C_{39}H_{50}O_{20}$	838.2895	838	289.50	sagittasine A	2
241	$C_{39}H_{50}O_{20}$	838.2895	838	289.50	3′-hydroxyicariine-3-O-rha-rha-7-O-glc	3
242	$C_{39}H_{50}O_{20}$	838.2895	838	289.50	epimedin A isomer	28
243	$C_{38}H_{50}O_{21}$	842.2845	842	284.50	dihydrodemethylicaritin-7-O-glc-3-O-glc-glc	3
244	$C_{40}H_{50}O_{20}$	850.2895	850	289.50	anhydroicaritin-3-O-rha(OAc)-xyl-7-O-glc	3
245	$C_{40}H_{50}O_{20}$	850.2895	850	289.50	anhydroicaritin-3-O-rha-7-O-glc-xyl(OAc)	3
246	$C_{40}H_{50}O_{20}$	850.2895	850	289.50	anhydroicaritin-3-O-rha(OAc)-xyl-7-O-glc isomer	3

247	$C_{40}H_{50}O_{20}$	850.2895	850	289.50	demethylanhydroicaritin-3-O-rha-rha-7-O-glc(OAc)	3
248	$C_{39}H_{50}O_{21}$	854.2845	854	284.50		28
249	$C_{41}H_{52}O_{20}$	864.3052	864	305.20	anhydroicaritin-3-O-rha-rha-7-O-glc(OAc)	3
250	$C_{40}H_{50}O_{21}$	866.2845	866	284.50	demethylanhydroicaritin-3-O-rha-glc(OAc)-7-O-glc	3
251	$C_{41}\ H_{52}O_{21}$	880.3001	880	300.10	korepimedoside C	11
252	$C_{41}H_{52}O_{21}$	880.3001	880	300.10	epimedin I	2
253	$C_{41}H_{52}O_{21}$	880.3001	880	300.10	anhydroicaritin-3-O-rha(1-4OAc)-7-O-(1-3)glc	3
254	$C_{42}H_{52}O_{21}$	892.3001	892	300.10	sempervirenoside A	41
255	$C_{42}H_{52}O_{21}$	892.3001	892	300.10	demethylicaritin-3-O- (6′′-O-acetyl)-glc -(1-3) -O- (4"-O-acetyl)-rha-7-O-glc	4
256	$C_{42}H_{52}O_{21}$	892.3001	892	300.10	epimedokoreanoside III	42
257	$C_{42}H_{52}O_{22}$	908.2950	908	295.00	demethylicaritin-3-O-(6′′-O-acetyl)-glc-(1-3)- O-(4"-O-acetyl) -rha-7-O-glc	18
258	$C_{42}H_{52}O_{22}$	908.2950	908	295.00	8-(3, 3-dimethylallyl)-4′, 5, 7-trihydroxyfla-vonol 3-[O-6-O-acetyl-β-D-glc-(1→3)-4-O-acetyl-α-L-rha] 7-(β-D-glc)	2
259	$C_{42}H_{52}O_{22}$	908.2950	908	295.00	demethylanhydroicaritin-3-O-rha(OAc)-glc(OAc)-7-O-glc	3
260	$C_{43}H_{54}O_{22}$	922.3107	922	310.70	caohuoside E	2
261	$C_{43}H_{54}O_{22}$	922.3107	922	310.70	epimedokoreanoside I	2
262	$C_{43}H_{54}O_{22}$	922.3107	922	310.70	epimedokoreanoside I isomer	3
263	$C_{43}H_{54}O_{22}$	922.3107	922	310.70	epimedokoreanoside I isomer	3
264	$C_{44}H_{54}O_{23}$	950.3056	950	305.60	demethylanhydroicaritin-3-O-rha(2OAc)-glc(OAc)-7-O-glc	3
265	$C_{44}H_{54}O_{23}$	950.3056	950	305.60	demethylanhydroicaritin-3-O-rha(2OAc)-glc(OAc)-7-O-glc isomer	3
266	$C_{44}H_{58}O_{23}$	954.3369	954	336.90		28
267	$C_{44}H_{58}O_{25}$	954.3369	954	336.90	demethylanhydroicaritin-3-O-rha-glc-7-O-sophoroside	3
268	$C_{45}H_{56}O_{23}$	964.3212	964	321.20	korepimedoside B	43
269	$C_{45}H_{56}O_{23}$	964.3212	964	321.20	epimedin K	2
270	$C_{45}H_{56}O_{23}$	964.3212	964	321.20	epimedin L	2
271	$C_{45}H_{56}O_{23}$	964.3212	964	321.20	caohuoside A	3
272	$C_{45}H_{56}O_{23}$	964.3212	964	321.20	caohuosideB	3
273	$C_{45}H_{56}O_{23}$	964.3212	964	321.20	korepimedoside C or isomer	3
274	$C_{45}H_{56}O_{23}$	964.3212	964	321.20	epemedin L isomer	14
275	$C_{45}H_{60}O_{23}$	968.3525	968	352.50	anhydroicaritin-3-O-rha-rha-7-O-rha-glc	3
276	$C_{45}H_{60}O_{24}$	984.3475	984	347.50	acuminatin	2
277	$C_{45}H_{60}O_{24}$	984.3475	984	347.50	acuminatoside	24

References

1. Wang, M.Q.; Peng, X.; Gan, Q.F. Studies on chemical constituents of *Epimedium brevicornum* Maxim. *Res. Pract. Chin. Med.* **2005,** *(02)*, 39-42.
2. Yuan, H.; Cao, S.P.; Chen, S.Y.; Guo, L.N.; Zheng, J.; Lin, R.C. Research progress on chemical constituents and quality control of *Epimedii Folium*. *Chin. Tradit. Herb. Drugs.* **2014,** *45(24)*, 3630-3640.
3. Zhao, H.Y.; Sun, J.H.; Fan, M.X.; Fan, L.; Zhou, L.; Li, Z.; Han, J.; Wang, B.R.; Guo, D.A. Analysis of phenolic compounds in Epimedium plants using liquid chromatography coupled with electrospray ionization mass spectrometry. *J.Chromatogr.A.* **2008,** *1190*,157-181.
4. Lv, L.; Zhang, H.; Zhao, L.; Jia, J.; Li, Y.Y.; Zhang, G.Q. RRLC-TOF/MS in rapid identification of 43 chemical constituents of epimedium. *Acad. J. Second Mil. Med. Univ.* **2011**, *32(03)*, 306-310.
5. Seelinger, G.; Merefort, I.; Schempp, C.M. Anti-oxidant, anti-inflammatory and anti-allergic activities of luteolin. *Planta. Med.* **2008**, *74(14)*, 1667-1677.
6. Huang, Y.L.; Ou, J.C.; Chen, C.F.; Chen, C.C. Three new 2-phenoxychromones from the leaves of *Epimedium Sagittatum. J. Nat. Prod.* **1993**, *56(2)*, 275-278.
7. Hartwig, U.A.; Maxwell, C.A.; Joseph, C.M.; Phillips, D.A. Chrysoeriol and luteolin released from alfalfa seeds induce nod genes in rhizobium meliloti. *Plant Physiol.* **1990**, *92*, 116-122.

8. Jiao, J.J.; Zhang, Y.; Liu, C.M.; Liu, J.E.; Wu, X.Q.; Zhang, Y. Separation and purification of tricin from an antioxidant product derived from bamboo leaves. *J. Agric. Food. Chem.* **2007**, *55*, 10086-10092.
9. Horio, T.; Kawabata, Y.; Takayama, T.; Tahara, S.; Kawabata, J.; Fukushi, I.Y.; Nishimura, H.; Mizutanil, J. A potent attractant of zoospores of aphanomyces cochlioides isolated from its host, *Spinacia oleracea*. *Experientia.* **1992**, *48*, 410-414.
10. Chen, C.C.; Huang, Y.L.; Sun, C.M.; Shen, C.C. New prenylflavones from the leaves of *Epimedium sagittatum*. *J. Nat. Prod.* **1996**, *59*, 412-414.
11. Sun, P.Y.; Wen, Y.; Xu, Y.; Pei, Y.P; Chen, Y.J.; Shimizu, N.; Takeda, T. The chemical constituents of Epimedium Koreanum Nakai. *Acta Pharm. Sin. B.* **1998**, *33(12)*, 919-922.
12. Li, W.K.; Pan, J.Q.; Lv, M.J.; Zhang, R.Y; Liao, M.C.; Xiao, P.G. Separation and structure of epimedokoreanin D. *Acta Pharm. Sin. B.* **1996**, *31(1)*, 29-32.
13. Chen, C.C.; Tsai, P.C.; Wei, B.L.; Chiob, W.F. 8-Prenylkaempferol suppresses inducible nitric oxide synthase expression through interfering with JNK-mediated AP-I pathway in murine macrophages. *Eur. J. Pharmacol.* **2008**, *590(1-3)*, 430-436.
14. Wang, Y.Q.; Guo, Z.M.; Jin, Y.; Zhang, X.L.; Wang, L.; Xue, X.Y.; Liang, X.M. Identification of prenyl flavonoid glycosides and phenolic acids in Epimedium koreanum Nakai by Q-TOF-MS combined with selective enrichment on "click oligo (ethylene glycol)" column. *J. Pharm. Biomed. Anal*. **2010**, *51(3)*, 606–616.
15. Jin, Q.H.; Lee, C.; Lee, J.W.; Yeon, E.T.; Lee, D.; Han, S.B.; Hong, J.T.; Kim, Y.; Lee, M.K; Hwang, B.Y. 2-Phenoxychromones and prenylflavonoids from Epimedium koreanum and their inhibitory effects on LPS-induced nitric oxide and interleukin-1β production. *J. Nat. Prod.* **2014**, *77(7)*, 1724-1728.
16. Li, H.F.; Guan, X.Y.; Yang, W.Z.; Liu, K.D.; Ye, M.; Sun, C.; Lu, S.; Guo, D.A. Antioxidant flavonoids from Epimedium wushanense. *Fitoterapia*. **2012** , *83(1)*, 44-48.
17. Sun, P.Y.; Chen, Y.; Shimizu, N.; Takeda, T. Studies on the contituents of epimedium koreanum III. *Chem. Pharm. Bull.* **1998**, 46(2), 355-358.
18. Gan, J.S.; Ma, Y.; Wang, Z.Y.; Liu, X.S.; Liu, Y. Analysis on chemical constituents of *Epimedii Herba* by UPLC-Q-TOF-MS. *Drugs & Clinic*. **2014**, 29(04), 349-352.
19. Guo, B.L.; Li, W.K.; Yu, J.G.; Xiao, P.G. A flavonol from *Epimedium Brevicornum*. *Phytochemistry.***1996**, *41(3)*, 991-992.
20. Wang, G.; Wang, C.Y.; Zhang, B.J.; Tian, Y. Tan, S.G.; Liu, T.X.; Sa, D.; Hou, J.; Peng, J.Y; Yao, J.H.; Ma, X.C. Rapid separation of flavonoids from hydrolysis products of *Epimedium koreanum*. *J. Liq. Chromatogr. Relat. Technol.* **2013**, *36*,1163-1176.
21. Cui, X.L.; Deng, L.L.; Huang, S.Y. Studies on chemical constituents in chloroform extraction of *Epimedium sagittatum*. *Chin. J. Exp. Tradit. Med. Formulae.* **2010**, *16(13)*,101-103.
22. Nakashima, K.; Miyashita, H.; Yoshimitsu, H.; Fujiwara, Y.; Nagai, R.; Ikeda, T. Two new prenylflavonoids from *Epimedii* Herba and their inhibitory effects on advanced glycation end-products. *J. Nat. Med.* **2010**, *70(2)*,290-295.
23. Hong, X.; Wang, X.C.; Yong, E.L.; Gong, Y.H. Determination of breviflavone A and B in Epimedium herbs with liquid chromatography-tandem mass spectrometry. *J Pharm Biomed Anal*. **2009**, 49(3), 853-857.
24. Yu, X.E.; Qin, J.P.; Li, J.C.; Huang, W.Z.; Wang, Z.Z.; Xiao, W. Rapid analysis on chemical constituents in Yinyanghuo Zonghuangtong capsule by UPLC /Q-TOF-MS/MS. *China J. Chin. Mater. Med.* **2008**, *590(1-3)*, 430-436.
25. Wang, Y.; Yuan, L.; Li, Y.B.; Zhang, Y.J. Analysis on chemical constituents of *Epimedii Folium* by UPLC-Q-TOF-MS. *Chin. Tradit. Herb. Drugs.* **2017**, *48(13)*, 2625-2631.
26. Li, W.K.; Zhang, R.Y.; Xiao, P.G. The structure of epimedokoreanin B and epimedokoreanin C. *Acta Pharm. Sin. B.* **1994**, *29(11)*, 835-839.
27. Toshio, F.; Taro, N. Seven prenylated flavonol glycosides from two Epimedium species. *Phytochemistry.***1988**, *27(1)*, 259-266.
28. Wu, C.S.; Zhang, J.L.; Zhou, T.H.; Guo, B.L.; Wang, Y.L.; Hou, J.F. Simultaneous determination of seven flavonoids in dog plasma by ultra-performance liquid chromatography-tandem mass spectrometry and its application to a bioequivalence study of bioactive components in Herba Epimedii and Er-Xian Decoction. *J. Pharm. Biomed. Anal.* **2011,** *54(1)*, 186-191.
29. Li, W.K.; Xiao, P.G.; Liao, M.C.; Zhang, R.Y. The structure of a new flavonoid glycoside from *Epimedium Koreanum* Nakai. *Chem. J. Chin. Univ.* **1995**, *16(10)*, 1575-1576.
30. Li, H. M.; Zhou, C.; Chen, C.H.; Li, R.T.; Lee, K.H. Flavonoids isolated from heat-processed *Epimedium Koreanum* and their anti-HIV-1 activities. *Helv. Chim. Acta.* **2015**, *98*, 1177-1187.
31. Pilepić, K.H.; Yang, Z.L.; Chen, J.; Chen, X.Q.; Wang, Y.; Zhao. J.; Mihaljević, S.; Li, S. P. Flavonoids in natural and tissue cultured materials of *Epimedium alpinum* identified by using UHPLC-Q-TOF-MS/MS. *Int. J. Mass Spectrom.* **2018**, *434*, 222-232.
32. Li, W.K.; Xiao, P.G.; Zhang, R.Y. A difuranoflavone from *Epimedium Koreanum*. *Phytochemistry.***1995**, *38(3)*, 807-808.
33. Mizuo, M.; Munekazu, I.; Toshiyuki, T.; Norio, S. New flavonol glycoside from the leaves of *Epimedium sempervirens*. *J. Nat. Prod.* **1990**, *53(3)*, 744-746.
34. Yu, C.Y.; Song, L.N.; Chen, G. Two new prenylflavonoids from *Epimedium sutchuenense*. *Chin. Chem. Lett.* **2009**, *20(7)*, 842-844.
35. Yan, W.M; Fu, Y.; Ma, Y.; Li, Y.W.; Zhang, X.Z.; Xin, F. Studies on flavones of *Epimedium bevicorum* Maxim. *China J. Chin. Mater. Med.* **1998**, *23(12)*, 31-32+59.

36. Li, F.; Liu, A.F. Studies on the isolation and structures of baohuoside II, III, IVand V. *Acta Pharm. Sin. B.* **1988**, *23(29)*, 672-681.
37. Mizuo, M.; Munekazu, I.; Toshiyuki, T.; Norio, S; Hirobumi, Y. Sutchuenoside A: a new kaempferol glycoside from the aerial parts of *Epimedium sutchuenense*. *J. Nat. Prod.***1991**, *54(5)*, 1427-1429.
38. Wang, G.J.; Tsai, T.H.; Lin, L.C. Prenylflavonol, acylated flavonol glycosides and related compounds from *Epimedium sagittatum*. *Phytochemistry.* **2007**, *68*, 2455-2464.
39. Sun, P.Y.; Chen, Y.J.; Wen, Y.; Pei, Y.P.; Liu, Z.H.; Yao, X.S.; Takeda, T.; Ogihara, Y. Structure determination of korepimedoside A and korepimedoside B from *Epimedium koreanum* Nakai. *Acta Pharm. Sin. B.* **1996**, *31(8)*, 602-606.
40. Yang, W.Z.; Qiao, X.; Bo, T.; Wang, Q.; Guo, D.A.; Ye, M. Low energy induced homolytic fragmentation of flavonol 3-O-glycosides by negative electrospray ionization tandem mass spectrometry. *Rapid. Commun. Mass Spectrom*. **2014**, *28(4)*, 385-395.
41. Mizuo, M.; Munekazu, I.; Toshiyuki, T.; Norio, S.; Tsutomu, N.; Akakira, I.M. A novel flavonol glycoside in the leaves of *Epimedium sempervirens*. *Chem. Pharm. Bull.* **1989**, 37(8), 2241-2242.
42. Xiang, D.S.; Li, W.; Ji, E.K.; Seo, Y.Y.; Jlin, Y.M.; Young, H.K. Preny-flavonoids from *Epimedium koreanum* Nakai and their soluble epoxide hydrolase and tyrosinase inhibitory activities. *Med. Chem. Res.* **2017**, *26(11)*, 2761-2767.
43. Li, J.Y.; Li, H.M.; Liu, D.; Chen, X.Q.; Chen, C.H.; Li, R.T. Three new acylated prenylflavonol glycosides from *Epimedium koreanum*. *Phytochem. Lett.* **2016**, *17*, 206-212.
